# Protein interaction networks characterizing the A549 cells Klotho transfected are associated with activated pro-apoptotic Bim and suppressed Wnt/β-catenin signaling pathway

**DOI:** 10.1038/s41598-024-52616-0

**Published:** 2024-01-25

**Authors:** Mitsuo Matsumoto, Naomi Ogawa, Tetsuya Fukuda, Yasuhiko Bando, Toshihide Nishimura, Jitsuo Usuda

**Affiliations:** 1 Department of Thoracic Surgery, Nippon Medical School, Tokyo, 113-8602 Japan; 2 Biosys Technologies, Inc, Tokyo, Tokyo, 153-8904 Japan; 3 Department of Translational Medicine Informatics, St. Marianna University School of Medicine, Kawasaki, Kanagawa 216-8511 Japan

**Keywords:** Biochemistry, Cancer, Cell biology, Computational biology and bioinformatics, Molecular biology, Systems biology, Diseases, Oncology

## Abstract

Invasive assays and lung tumor-bearing mice models using a human lung adenocarcinoma cell line A549 cells transfected with the Klotho (KL) gene, A549/KL cells, have confirmed that KL suppresses invasive/metastatic potential. This study aimed to identify the co-expression protein networks and proteomic profiles associated with A549/KL cells to understand how Klotho protein expression affects molecular networks associated with lung carcinoma malignancy. A two-step application of a weighted network correlation analysis to the cells’ quantitative proteome datasets of a total of 6,994 proteins, identified by mass spectrometry-based proteomic analysis with data-independent acquisition (DIA), identified one network module as most significantly associated with the A549/KL trait. Upstream analyses, confirmed by western blot, implicated the pro-apoptotic Bim (Bcl-2-like protein 11) as a master regulator of molecular networks affected by Klotho. GeneMANIA interaction networks and quantitative proteome data implicated that Klotho interacts with two signaling axes: negatively with the Wnt/β-catenin axis, and positively by activating Bim. Our findings might contribute to the development of future therapeutic strategies.

## Introduction

Lung cancer is the most common cancer worldwide, accounting for 2.5 million new cases and 1.5 million deaths in 2019, among which non-small-cell lung carcinoma predominantly accounts for more than 80%^1^. Even though many patients receive early diagnoses with low-dose spiral computed tomography, the 5-year survival rate remains less than 20% among patients who received chemo-, targeted, and immunotherapies. These deaths are thought to be because either those patients were diagnosed at an advanced stage or they received an early diagnosis that was incorrect^1^.

The *klotho* (*KL*) gene encodes a type-I membrane protein related to beta-glucuronidases and has three subfamilies: α-Klotho, β-Klotho (KLB), and γ-Klotho. *KL* refers to α-Klotho, which was first identified in a study of *KL*-deficient mice that developed multiple premature aging syndromes, contrasted with *KL*-overexpression associated with the extended lifespan of the mice^2,3^. Thus, *KL* received great attention as a new anti-aging gene that critically regulates aging and the development of age-related diseases. On the other hand, the *KL* gene has been recently highlighted in its association with tumor growth and invasion in various cancers, including breast, pancreatic, ovarian, lung, colon, and melanoma^4^. It is known that *KL* is involved in various biological processes and inhibits the insulin-like growth factor (IGF-1) signaling pathway^5^, which may be associated with malignancies of non-small-cell lung carcinoma (NSCLC) and small-cell carcinoma (SCLC)^6^.

Recently, Zhou et al. found that the concentrations of serum KLB were considerably higher in patients with NSCLC than in the control group and that KLB expression was significantly increased in patients after chemotherapy and epidermal growth factor receptor tyrosine kinase inhibitor (EGFR-TKI) targeted therapy as well as showing good correlation with progression-free survival (PFS) and overall survival (OS)^7^. Chen et al. constructed human lung cancer A549 cell lines transfected with *Klotho* or *Klotho-specific* shRNAs to investigate overexpression or knockdown Klotho in vitro. They suggested that Klotho can inhibit proliferation and increase apoptosis of A549 cells, partly due to the inhibition of IGF-1/insulin pathways and involving regulation of BAX/BCL2 expression (apoptosis-related genes)^8^. The authors suggested that the *KL* gene can potentially suppress tumors.

As described previously, we established an A549/KL cell line with stable and high Klotho protein expression by transfecting green fluorescent protein (GFP)-*klotho* plasmids into lung adenocarcinoma A549 cells^9^. It has been demonstrated in the A549/KL cell line that highly expressed *KL* significantly suppressed N-cadherin (CDH2) expression, an endothelial mesenchymal transition (EndMT)-related protein, which is also a favorable prognostic factor in lung cancer^10,11^. This observation formed the basis of a hypothesis that the *KL* gene induces inhibition of metastasis and invasiveness of lung cancer cells. Clinically, we sought to learn how the *KL* gene and protein affect malignancies, to develop treatment strategies to improve outcomes of patients with lung cancer.

A critical need is to identify molecular networks induced by the *KL* gene and its upstream regulators, which might help elucidate the mechanisms underlying the suppression of cancer malignancies. Recent advances in mass spectrometry (MS) have made MS-based proteomics much more powerful for use in shotgun protein sequencing and quantitative analysis of proteins expressed in clinical specimens than in the past. Quantitative proteome data can be used to identify key disease-related proteins and therapeutic targets in oncology^12^. We have used a high-performance mass spectrometer operated in the data-independent acquisition (DIA) mode to perform label-free ion intensity-based quantitative proteomics. We used the universal automated software suite DIA-NN (DIA-neural networks), which is particularly useful for performing high-throughput proteomics by enabling fast and reliable protein identification^13^.

This study aimed to identify co-expression protein networks associated with A549/KL cells, compared with those of A549 cells, to understand how Klotho protein expression affects molecular networks associated with the malignant nature of lung carcinoma cells. Weighted gene co-expression network analysis (WGCNA), an unsupervised clustering method based on correlation network expression^14,15^ was applied to quantitative proteome datasets. Then, Ingenuity Pathway Analysis was used to perform upstream analysis^16^ of data-driven protein co-expression networks, and GeneMANIA^17^ was used to identify interaction networks of proteins expressed characteristically in the A549 and A549/KL cells, respectively.

## Results

### Invasion assay

**Figure 1:**
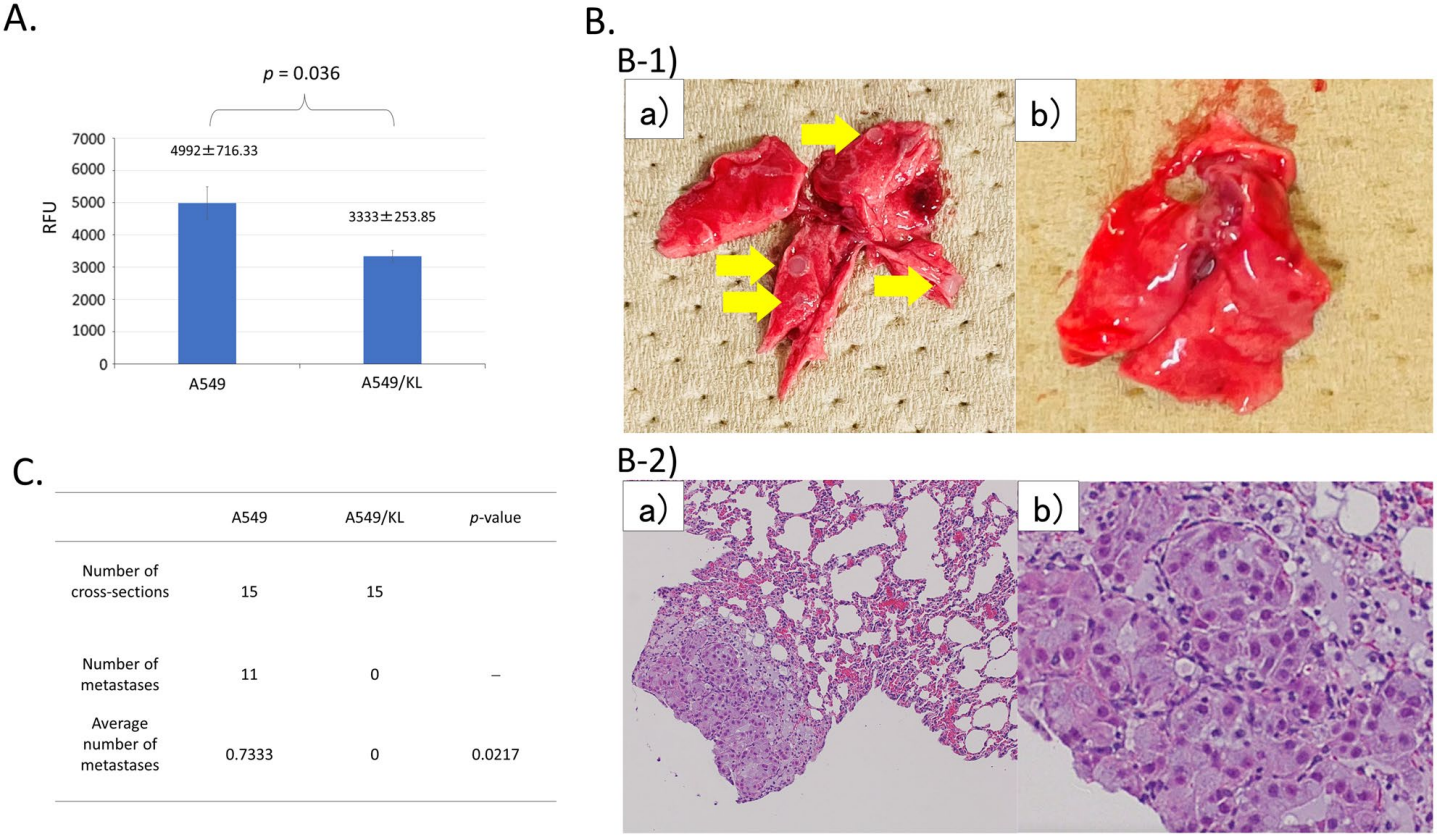
(**A**) Invasion ability of A549 and A549/KL cells determined using a cell invasion assay kit. Three samples of each cell were quantified. The mean number of invasive cells was compared, indicating a statistically significant reduction in invasive ability for the A549/KL groups (*p* = 0.036). (**B**) Representative extracted lungs of mice. (**B-1**) The A549 cell-injected mice developed metastases in their lungs with multiple nodules on the surface of the lungs (**a**), but the A549/KL mice never developed metastases in their lungs (**b**). The lung nodule is indicated with an arrow. (**B-2**) Representative images of hematoxylin and eosin (H&E) staining of the lung sections of the A549 group. Image b is an enlarged view of image a. (**C**) A549 and A549/KL cells (5.0 × 10^6^ cells) were injected into the tail veins of mice (three mice/group) to generate lung tumor metastases. Fifteen sections of lung tissue from three mice, five sections of lung tissue per mouse, were evaluated. Comparison of the mean number of metastases per cross-section showing the reduced metastatic ability of the A549/KL groups with a statistical significance of *p* = 0.0217.

A CytoSelect 24-well cell invasion assay kit was used to evaluate the invasive ability of the A549 and A549/KL cells. A fluorescence method was used to quantify the infiltrating cells, and the average values were compared. The results showed that the invasive capacity was considerably reduced for the A549/KL cells than for the parental A549 cells (Fig. 1A).

### Metastasis model of tumor-bearing mice

**Figure 2:**
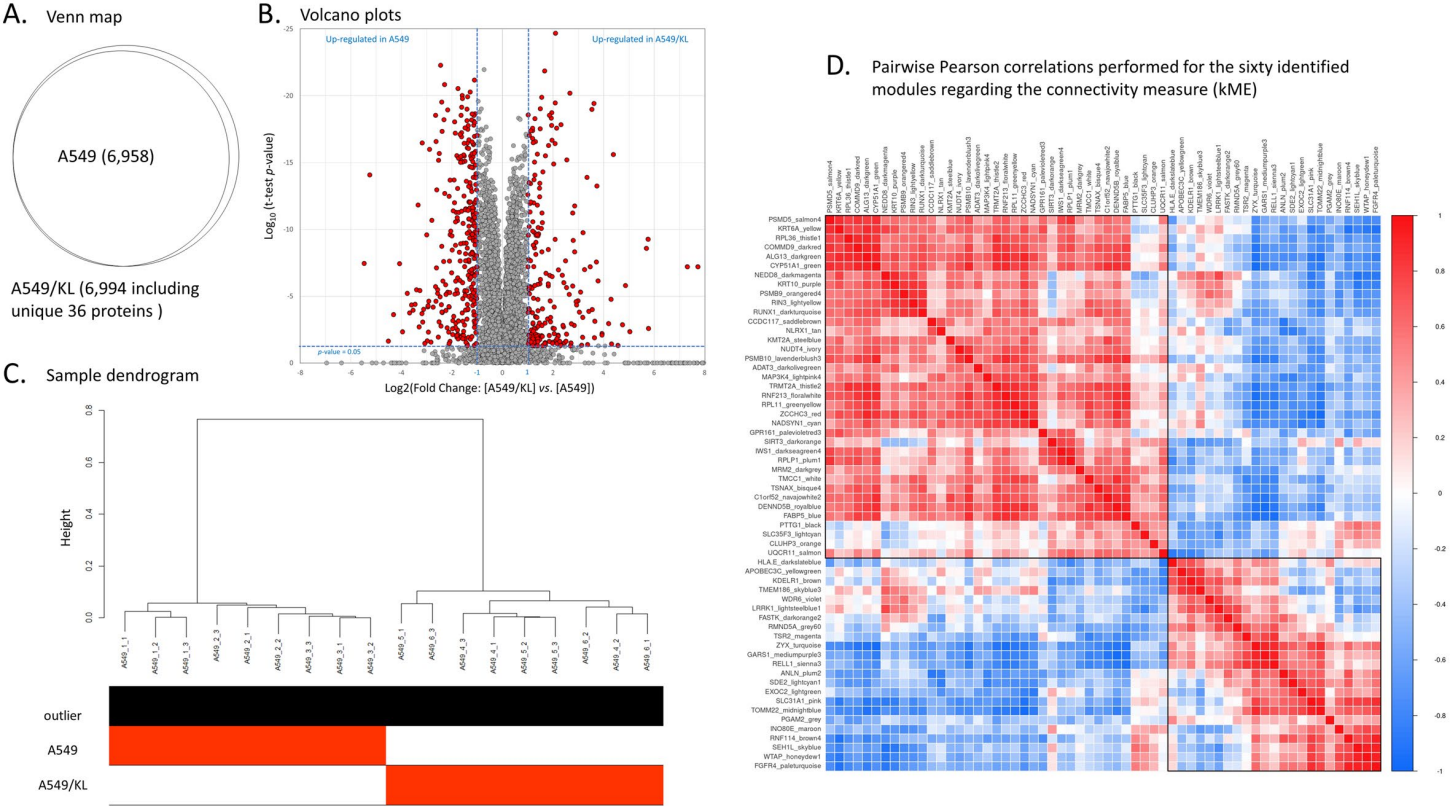
(**A**) Venn map of the proteins identified by mass spectrometry-based analysis and (**B**) their volcano plots, where proteins with *p* value < 0.05 and |log2 (Fold Change: [A549/KL]*/*[A549])|> 1 are indicated by red-filled circles. (**C**) Sample dendrogram and trait heatmap for the A549 (*n* = 3) and A549/KL (*n* = 3) with the triplicate measurements, which were constructed by the Euclidian distance-based network used in the weighted gene co-expression network analysis (WGCNA) software. (**D**) Pairwise Pearson correlations performed for the 60 identified modules regarding the connectivity measure (kME) of the module eigen-protein (correlation coefficient: Pearson’s *r*; heatmap order: eigenvectors; agglomeration method: complete; the number of clusters: 2).

A lung tumor-bearing mice model was used to determine if high *KL* gene expression can suppress lung metastasis. A549 or A549/KL cells were injected into the tail vein of three mice each. After 8 weeks, the lungs were removed and pathologically diagnosed (Fig. 1B). Metastatic tumors of A549 were observed macro- and microscopically, as shown in Fig. 1B-1 and B-2, respectively, whereas no metastasis was observed in the A549/KL cells. There was a significant difference in the frequency of lung metastasis between the A549 and A549/KL cells; no lung metastasis occurred in mice that received A549/KL cells, confirming that *KL* expression suppressed cancer cell metastasis (Fig. 1C).

### MS-based proteome datasets of A549 and A549/KL cells

In-depth MS-based proteomic analysis was performed for the A549 adherent cells and A549/KL cells transfected with the *KL* gene (*n* = three samples each), resulting in a total of six samples. Each sample contained approximately 2 × 10^6^ cells. A total of 6,994 proteins were identified, of which 6,958 (99.49%) were commonly expressed in the A549 and A549/KL cells, and only 36 (0.51%) proteins were unique to the A549/KL cells, a finding that showed an extremely high protein expression similarity between the cell lines (Fig. 2A). Volcano plots of the protein expression were generated using SimpliFi™ software (PROTIFI, Farmingdale, NY, USA; https://simplifi.protifi.com/) (Fig. 2B), exhibiting highly different expression levels. In the analysis, upregulated expressions of 215 and 302 proteins (*p* value < 0.05 and |log2 (Fold Change: [A549/KL]/[A549])|> 1) were shown for the A549/KL and A549 cells, respectively.

### Identification of co-expression protein networks by WGCNA

Following hierarchical clustering of the samples based on protein abundance (Fig. 2C), we used dynamic tree-cut, block-wise, and manual hybrid methods to perform a WGCNA analysis ^14^ utilizing the adjacency of an unsigned network with a soft threshold power of 10 (which was selected to approximate a scale-free topology), a minimum module size of 10, and a module detection sensitivity (*deepSplit*) of 4 (Figure S1). Then, we merged highly correlated modules to obtain the total protein cluster dendrogram. Correlations between the resultant modules and traits were obtained to identify protein modules that were significantly associated with the respective traits. Pairwise correlations between the sixty WGCNA modules identified were obtained regarding the connectivity measure, kME, of the module eigen-protein (Fig. 2D). A module is significant to a trait when its correlation *r* is > 0.5 and the *q* value is < 0.05.

**Figure 3:**
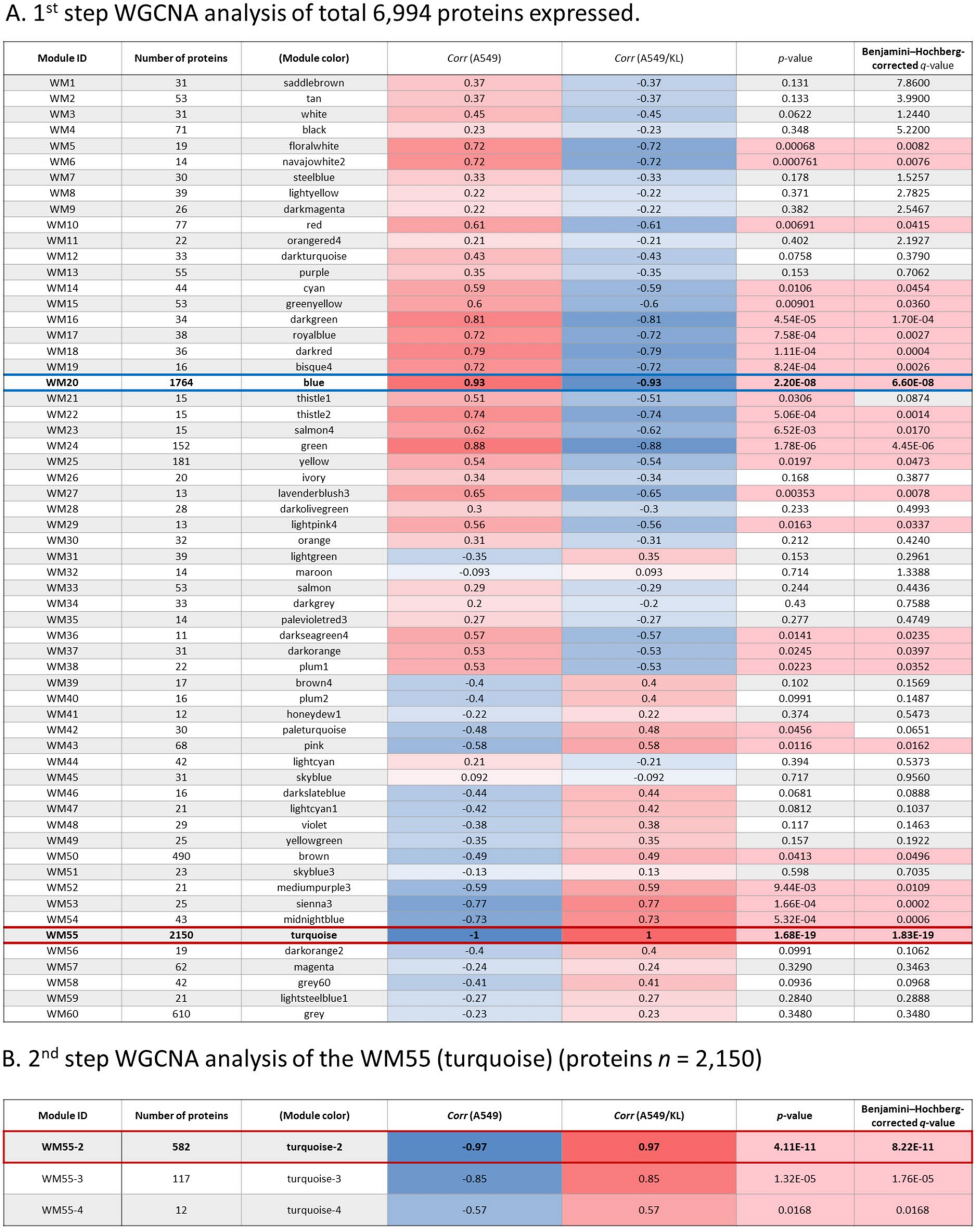
Module-trait relationships. (**A**) the first step of the WGCNA analysis of 6,994 of proteins expressed. (**B**) the second step of the WGCNA analysis of the WM55 (turquoise) module (the number of proteins *n* = 2,150). Benjamini–Hochberg-corrected *q*-values were presented as a multiple statistical test.

A module-trait relationship obtained for sixty protein network modules (Fig. 3A) showed that 20 and 5 modules were significant to the A549 and A549/KL traits, respectively. We focus on modules significantly associated with the A549/KL trait. The WM55 (turquoise) module was found to have the highest significant association with the A549/KL trait (*r* = 1.0; *q* value = 1.01 × 10^−17^). However, the WM55 (turquoise) module has 2,150 module member proteins, which was too many to conduct further network analysis. Then, we decided to apply the second WGCNA analysis to those modules. Finally, the WM55-2 (turquoise-2) module consisting of 582 member proteins was identified as by far the most highly significant to the A549/KL trait (*r* = 0.97 and *q* value = 8.22 × 10^−11^) (Fig. 3B). The module membership of module proteins (kME *vs*. gene/protein significance) for the WM55-2 (turquoise-2) module showed a qualified correlation and a significant association with the A549/KL trait (Supplementary Figure S2).

Using the Search Tool for the Retrieval of Interacting Genes/Proteins (STRING) database version 11.5 (https://string-db.org/)^18^, the protein–protein interaction (PPI) networks of the WM55-2 (turquoise-2) module was reconstructed with Cytoscape (version 3.9.1) software (Institute for Systems Biology, Seattle, WA, USA: https://cytoscape.org/) (Figure S3). The *cytoHubba* plugin with maximal clique centrality was used to calculate the top ten hub proteins^19^. In this data-driven protein co-expression network, hub proteins are denoted in red-to-orange fill colors.

### Upstream analysis by ingenuity pathway analysis (IPA)

Upstream analysis for the WM55-2 (turquoise-2) module was performed with IPA software (http://www.ingenuity.com)^16^. The top 20 master regulators (genes, RNAs, and proteins) together with participating regulators, canonical pathways, and regulator effects predicted for the turquoise-2 module are listed in Supplementary Table S1.

Top upstream regulators predicted to be activated (*z* > 2.0) include *ERN1* (*IRE1*), *MYC*, *EGFR*, *NFE2L2*, *XBP1*, *IL4*, *FN1*, *TGFB1*, and *CEBPB* in the significance order of the overlap* p*-value. Top highly activated causal networks (*z* > 4.0) include *CXCL14*, *BBC3* (BCL2 binding component 3, also known as *JFY-1* or *Puma*), *Rasgrp* (*RASGRP1-3*), *PRRG4*, BCL2-like 11(*BCL2L11*, also known as apoptosis facilitator, *Bim*), *RAS*, and *Egfr-Erbb2* in the significance order of the network bias-corrected *p*-value. Thus, those regulators might be categorized into carcinogenesis or pro-apoptotic functions. NFE2L2 is the master regulator of hypoxia and co-activation of ERN1(IRE1) and XBPI is associated not only with unfolding protein response (UPR) in ER stress (the inositol requiring enzyme 1-alpha (IRE1α)/X-box binding protein 1 pathway), but also with cell death and/or the apoptotic process^20^. Overexpression of fibronectin 1 (FN1) is associated with tumor progression by promoting proliferation, invasion, and metastasis^21^. Most of the top regulator effects predicted were annotated characteristically as apoptosis of carcinoma cell lines, including lung cancer (Table S1). Therein, it should be noted that only Bim and Puma involve apoptosis regulator BAX (BAX) and Bcl-2-related ovarian killer protein (BOK) as their participating regulators among the top causal networks (Table 1). Indeed, our proteomic analysis captured BAX and upregulated BOK in the A549/KL cells (Table 2), implicating that both Bim and Puma are the most likely candidates for a master regulator affected by the *KL* gene.

**Table 1:** The master regulator and causal networks of BBC3 (Puma) and BCL2L11 (Bim) predicted to be highly activated for the WM55-2 (turquoise-2) module (extracted from Table S1). Only Puma and Bim among the top master regulators include the key participating apoptosis regulators, BAX, BOK, and BAK1, which expressions were observed in the A549/KL cells (see Table 2).

Master regulator	Participating regulators	Activation *z*-value	*p* value of overlap	Network bias corrected *p* value
BBC3	26sProteasome,AKT1,Ap1,APEX1,AR,ATF4,BAK1,BAX,BBC3,BCL2,BCL2L1,BOK,CASP1,CASP3,CASP9,caspase,CDK2,CDKN1B,CEBPA,CEBPB,DDIT3,DICER1,EIF2AK2,ERN1,ESR1,IKBKB,IRF3,JUN,MAPK1,Mek,MMP9,PCYT1A,PTEN,PTK2B,RNASEL,SP1,SRC,XBP1	4.835	1.61E-14	0.0001
BCL2L11	26sProteasome,Ap1,APEX1,AR,ATF4,BAK1,BAX,BCL2,BCL2L11,BOK,CASP1,CASP3,caspase,CDK2,CEBPA,CEBPB,DDIT3,DICER1,EIF2AK2,ERN1,ESR1,IKBKB,IRF3,JUN,MAPK1,Mek,PCYT1A,PTEN,RNASEL,SP1,SRC,XBP1	4.906	3.94E-14	0.0001

**Table 2:** (A) The expressions of twenty-three adhesion- and apoptosis-related proteins with |Log2(FC)|> 0.585: i.e. |FC|> 1.5 (A549/KL vs A549). (B) Representative expressions of Klotho and key proteins associated with cancer- and apoptosis-related pathways. (C) Main proteins participating in the Wint/β-catenin and the Wnt signaling pathways. (D) Matrix metalloproteinase genes/proteins.

Accession number	Gene name	Description	Wilcoxon *p*-value	Log2 (FC) (|Log2(FC)|> 0.585: i.e. |FC|> 1.5)
A. Adhesion- and apoptosis-related proteins				
P06731	CEACAM5, CEA	Carcinoembryonic antigen-related cell adhesion molecule 5	NCO	− 12.272
P40199	CEACAM6, NCA	Carcinoembryonic antigen-related cell adhesion molecule 6	NCO	− 5.691
P13688	CEACAM1, BGP, BGP1	Carcinoembryonic antigen-related cell adhesion molecule 1	NCO	− 4.421
P16422	EPCAM, GA733-2, M1S2, M4S1, MIC18, TACSTD1, TROP1	Epithelial cell adhesion molecule	2.06E − 05	− 2.543
Q0VAQ4	SMAGP	Small cell adhesion glycoprotein	NCO	− 2.384
P12830	CDH1, CDHE, UVO	Cadherin-1	2.06E − 05	− 2.086
P26232	CTNNA2, CAPR	Catenin alpha-2	4.11E − 05	− 2.084
Q12864	CDH17	Cadherin-17	NCO	− 1.921
Q5H9F3	BCORL1	BCL-6 corepressor-like protein 1	0.08547	− 1.171
P35222	CTNNB1, CTNNB, OK/SW-cl.35, PRO2286	Catenin beta-1	2.06E − 05	− 0.901
Q9HB09	BCL2L12, BPR	Bcl-2-like protein 12	0.0002	0.612
Q9BY67	CADM1, IGSF4, IGSF4A, NECL2, SYNCAM, TSLC1	Cell adhesion molecule 1	2.06E − 05	0.629
Q07820	MCL1, BCL2L3	Induced myeloid leukemia cell differentiation protein Mcl-1	2.06E − 05	0.689
Q92934	BAD, BBC6, BCL2L8	Bcl2-associated agonist of cell death	4.11E − 05	0.724
P50895	BCAM, LU, MSK19	Basal cell adhesion molecule	2.06E − 05	0.981
Q92823	NRCAM, KIAA0343	Neuronal cell adhesion molecule	2.06E − 05	1.277
Q9HC56	PCDH9	Protocadherin-9	2.06E − 05	1.328
Q9UN75	PCDHA12	Protocadherin alpha-12	8.74E − 05	1.353
Q14517	FAT1, CDHF7, FAT	Protocadherin Fat 1	2.06E − 05	1.635
Q8NFZ8	CADM4, IGSF4C, NECL4, TSLL2	Cell adhesion molecule 4	NCO	2.073
Q9Y653	ADGRG1, GPR56, TM7LN4, TM7XN1, UNQ540/PRO1083	Adhesion G-protein coupled receptor G1	2.06E − 05	2.498
Q9P266	JCAD, KIAA1462	Junctional cadherin 5-associated protein	NCO	6.954
Q9UMX3	BOK, BCL2L9	Bcl-2-related ovarian killer protein	NCO	15.293

Accession number	Gene name	Description	Wilcoxon *p* value	Log2(FC)
B. Klotho and key proteins associated with cancer- and apoptosis-related pathways				
P46527	CDKN1B, KIP1	Cyclin-dependent kinase inhibitor p27,p27Kip1	2.06E − 05	− 1.373
P12931	SRC, SRC1	Proto-oncogene tyrosine-protein kinase Src	2.06E − 05	− 0.547
P99999	CYC, CYCS	Cytochrome c	2.06E − 05	− 0.527
Q07812	BAX, BCL2L4	Apoptosis regulator BAX	1.44E − 04	− 0.287
Q8NEB9	PIK3C3, VPS34	Phosphatidylinositol 3-kinase catalytic subunit type 3, PI3-kinase type 3, PI3K type 3	2.00E − 03	− 0.258
P55061	BI1, tEGT, TMBIM6	Bax inhibitor 1	7.10 − 03	− 0.229
Q07817	BCL2L, BCLX, BCL2L1	Bcl-2 like protein 1	0.0157	− 0.197
Q13158	FADD, GIG3, MORT1	FAS-associated death domain protein	0.129	− 0.147
P17252	PKCA, PRKACA, PRKCA	Protein kinase C alpha type, PKC-A, PKC-alpha	3.91E − 04	− 0.147
P28482	ERK2, PRKM1,PRKM2,MAPK1	Mitogen-activated protein kinase 1	0.0122	0.055
P22455	FGFR4, JTK2, TKF	Fibroblast growth factor receptor 4	0.0385	0.155
P31749	AKT1, PKB, RAC	0RAC-alpha serine/threonine-protein kinase	0.068	0.167
P04049	RAF, RAF1	RAF proto-oncogene serine/threonine-protein kinase	0.068	0.218
Q16611	BAK, BCL2L7, CDN1, BAK1	Bcl-2 homologous antagonist/killer	3.91E − 04	0.415
O14727	APAF1、KIAA0413	Apoptotic protease-activating factor 1	0.0568	0.521
P19174	PLCG1	Phospholipase C-gamma-1 (PLC-gamma-1)	2.06E − 05	0.539
P00533	EGFR,ERBB, ERBB1, HER1	Epidermal growth factor receptor	2.06E − 05	0.559
P08581	MET	Hepatocyte growth factor receptor	2.06E − 05	0.594
O00220	TNFRSF10A, APO2, DR4, TRAILR1	Tumor necrosis factor receptor superfamily member 10A	2.06E − 05	0.719
P09038	FGF2, FGFB	Fibroblast growth factor 2	2.06E − 05	0.960
P11362	FGFR1, BFGFR, CEK, FGFBR, FLG, FLT2, HBGFR	Fibroblast growth factor receptor 1	2.06E − 05	1.246
O14763	TNFRSF10B, DR5, KILLER, TRAILR2, TRICK2, ZTNFR9, UNQ160/PRO186	Tumor necrosis factor receptor superfamily member 10B	2.06E − 05	1.401
Q9UEF7	KL	Klotho	0.0002	2.008
P08670	VIM	Vimentin	2.06E − 05	2.789
P37275	ZEB1, AREB6, TCF8	Zinc finger E-box-binding homeobox 1	NCO	4.5122
Q13950	RUNX2, AML3, CBFA1, OSF2, PEBP2A	Runt-related transcription factor 2	NCO	18.460
P29279	CCN2, CTGF, HCS24, IGFBP8	CCN family member 2	NCO	19.209
C. Main proteins participating in the Wint/β-catenin and the Wnt signaling pathways				
Major components participating in the Wint/β-catenin pathway				
O94907	DKK1	Dickkopf-related protein 1	2.00E − 04	− 2.047
P35222	CTNNB1	Catenin beta-1	2.06E − 05	− 0.901
Q9BXY4	RSPO3	R-spondin-3	2.06E − 05	− 0.559
Q9BXB1	LGR4	Leucine-rich repeat-containing G-protein coupled receptor 4	0.0200	− 0.226
Q5T9L3	WLS	Protein wntless homolog	0.1112	− 0.072
Q14332	FZD2	Frizzled-2	NCO	2.741
Q13467	FZD5	Frizzled-5	0.0313	0.203
O60353	FZD6	Frizzled-6	2.06E − 05	0.647
O75084	FZD7	Frizzled-7	3.91E − 04	0.815
O75197	LRP5	Low-density lipoprotein receptor-related protein 5	0.0014	0.496
O75581	LRP6	Low-density lipoprotein receptor-related protein 6	2.06E − 05	0.743
O95996	APC2	Adenomatous polyposis coli protein 2	0.0951	0.117
P49841	GSK3B	Glycogen synthase kinase-3 beta	8.23E − 05	0.190
Q99081	TCF12	Transcription factor 12	0.1701	− 0.515
Q9UGU0	TCF20	Transcription factor 20	0.3029	− 0.018
Q9BQ70	TCF25	Transcription factor 25	0.3024	− 0.039
O14641	DVL2	Segment polarity protein dishevelled homolog DVL-2	0.0568	0.329
Q9ULT6	ZNRF3	E3 ubiquitin-protein ligase ZNRF3	6.12E − 04	0.947
Q14517	FAT1	Protocadherin Fat 1	2.06E − 05	1.635
Q9H237	PORCN	Protein-serine O-palmitoleoyltransferase porcupine	NCO	3.988
Other proteins participating in the Wnt signaling pathways				
P49407	ARRB1	Beta-arrestin-1	2.00E − 04	− 2.777
P12830	CDH1	Cadherin-1	2.06E − 05	− 2.086
Q12864	CDH17	Cadherin-17	NCO	− 1.921
P35221	CTNNA1	Catenin alpha-1	2.06E − 05	− 1.217
Q6ZRS2	SRCAP	Helicase SRCAP	2.06E − 05	− 1.003
P48454	PPP3CC	Serine/threonine-protein phosphatase 2B catalytic subunit gamma isoform	NCO	− 0.817
Q15797	SMAD1	Mothers against decapentaplegic homolog 1	0.0708	− 0.812
O60907	TBL1X	F-box-like/WD repeat-containing protein TBL1X	NCO	− 0.641
Q86WJ1	CHD1L	Chromodomain-helicase-DNA-binding protein 1-like	2.06E − 05	− 0.521
Q14573	ITPR3	Inositol 1,4,5-trisphosphate receptor type 3	2.06E − 05	− 0.387
P56524	HDAC4	Histone deacetylase 4	0.4726	− 0.369
P32121	ARRB2	Beta-arrestin-2	2.82E − 03	− 0.330
Q96GM5	SMARCD1	SWI/SNF-related matrix-associated actin-dependent regulator of chromatin subfamily D member 1	4.11E − 05	− 0.298
Q13547	HDAC1	Histone deacetylase 1	2.06E − 05	− 0.291
Q9BY41	HDAC8	Histone deacetylase 8	1.38E − 03	− 0.222
Q08209	PPP3CA	Protein phosphatase 3 catalytic subunit alpha	2.47E − 04	− 0.210
Q9ULG1	INO80	Chromatin-remodeling ATPase INO80	0.1290	− 0.207
Q9NRZ9	HELLS	Lymphoid-specific helicase	4.11E − 05	− 0.166
O15379	HDAC3	Histone deacetylase 3	0.1933	− 0.148
P17252	PRKCA	Protein kinase C alpha type	3.91E − 04	− 0.147
Q13485	SMAD4	Mothers against decapentaplegic homolog 4	0.1933	− 0.144
Q14527	HLTF	Helicase-like transcription factor	0.0014	− 0.141
P63098	PPP3R1	Calcineurin subunit B type 1	0.2181	− 0.133
Q96BJ3	AIDA	Axin interactor, dorsalization-associated protein	0.0568	− 0.127
Q92922	SMARCC1	SWI/SNF complex subunit SMARCC1	0.0039	− 0.121
Q05655	PRKCD	Protein kinase C delta type	0.0470	− 0.079
Q15172	PPP2R5A	Serine/threonine-protein phosphatase 2A 56 kDa regulatory subunit alpha isoform	0.0951	− 0.073
P62714	PPP2CB	Serine/threonine-protein phosphatase 2A catalytic subunit beta isoform	0.3652	− 0.062
Q14738	PPP2R5D	Serine/threonine-protein phosphatase 2A 56 kDa regulatory subunit delta isoform	0.0094	− 0.061
Q09472	EP300	Histone acetyltransferase p300	0.2447	− 0.046
Q9BZK7	TBL1XR1	F-box-like/WD repeat-containing protein TBL1XR1	0.4657	− 0.037
Q12824	SMARCB1	SWI/SNF-related matrix-associated actin-dependent regulator of chromatin subfamily B member 1	0.2729	− 0.027
Q969G3	SMARCE1	SWI/SNF-related matrix-associated actin-dependent regulator of chromatin subfamily E member 1	0.5000	− 0.011
P68400	CSNK2A1	Casein kinase II subunit alpha	0.1487	0.032
P51531	SMARCA2	Probable global transcription activator SNF2L2	0.2447	0.039
Q92793	CREBBP	CREB-binding protein	0.3365	0.040
O60264	SMARCA5	SWI/SNF-related matrix-associated actin-dependent regulator of chromatin subfamily A member 5	0.0568	0.049
P67775	PPP2CA	Serine/threonine-protein phosphatase 2A catalytic subunit alpha isoform	0.0122	0.054
P19784	CSNK2A2	Casein kinase II subunit alpha'	0.1701	0.055
Q14571	ITPR2	Inositol 1,4,5-trisphosphate receptor type 2	NCO	0.066
Q04724	TLE1	Transducin-like enhancer protein 1	0.3332	0.082
P67870	CSNK2B	Casein kinase II subunit beta	0.0200	0.096
Q92769	HDAC2	Histone deacetylase 2	1.44E − 04	0.121
Q14643	ITPR1	Inositol 1,4,5-trisphosphate receptor type 1	0.0680	0.123
Q9BRQ0	PYGO2	Pygopus homolog 2	0.0200	0.151
Q9NYQ6	CELSR1	Cadherin EGF LAG seven-pass G-type receptor 1	0.0385	0.171
Q15147	PLCB4	1-phosphatidylinositol 4,5-bisphosphate phosphodiesterase beta-4	0.1487	0.186
Q13362	PPP2R5C	Serine/threonine-protein phosphatase 2A 56 kDa regulatory subunit gamma isoform	2.06E − 05	0.235
O43318	MAP3K7	Mitogen-activated protein kinase kinase kinase 7	0.05675	0.244
P28370	SMARCA1	Probable global transcription activator SNF2L1	2.06E − 05	0.279
Q9NZC9	SMARCAL1	SWI/SNF-related matrix-associated actin-dependent regulator of chromatin subfamily A-like protein 1	0.0470	0.379
Q04726	TLE3	Transducin-like enhancer protein 3	2.06E − 05	0.393
Q9Y6M4	CSNK1G3	Casein kinase I isoform gamma-3	2.06E − 05	0.434
P29992	GNA11	Guanine nucleotide-binding protein subunit alpha-11	2.06E − 05	0.482
P50148	GNAQ	Guanine nucleotide-binding protein G(q) subunit alpha	2.06E − 05	0.517
P62873	GNB1	Guanine nucleotide-binding protein G(I)/G(S)/G(T) subunit beta-1	2.06E − 05	0.524
Q92925	SMARCD2	SWI/SNF-related matrix-associated actin-dependent regulator of chromatin subfamily D member 2	4.11E − 05	0.528
P62879	GNB2	Guanine nucleotide-binding protein G(I)/G(S)/G(T) subunit beta-2	2.06E − 05	0.612
P16435	POR	NADPH–cytochrome P450 reductase	2.06E − 05	0.648
Q9HAV0	GNB4	Guanine nucleotide-binding protein subunit beta-4	2.06E − 05	0.657
Q8NCF5	NFATC2IP	NFATC2-interacting protein	2.06E − 05	0.716
Q9UKB1	FBXW11	F-box/WD repeat-containing protein 11	0.1338	0.767
P16298	PPP3CB	Serine/threonine-protein phosphatase 2B catalytic subunit beta isoform	NCO	1.125
Q9HC56	PCDH9	Protocadherin-9	2.06E − 05	1.328
Q9UN75	PCDHA12	Protocadherin alpha-12	8.74E − 05	1.353
Q6STE5	SMARCD3	SWI/SNF-related matrix-associated actin-dependent regulator of chromatin subfamily D member 3	NCO	3.987
Q8TAQ2	SMARCC2	SWI/SNF complex subunit SMARCC2	NCO	15.764
D. Matrix metalloproteinase genes/proteins				
O60882	MMP20	Matrix metalloproteinase-20	NCO	− 1.456
P51511	MMP15	Matrix metalloproteinase-15	NCO	20.132

*NCO* no comparison object.

Fold change was calculated as [total ion intensity (A549/KL) + 1]/[total ion intensity (A549) + 1].

Canonical pathways predicted to be activated were the UPR, EIF2 signaling, NRF2-mediated oxidative stress response, insulin-secretion signaling pathway, pulmonary fibrosis idiopathy signaling pathway, regulation of the epithelial–mesenchymal transition (EMT) by growth factors pathway, and EGF signaling (Table S1).

The integrative causal networks of Bim and Puma (presented in Figure S4) constructed by IPA implicate that Bim and Puma are unphosphorylated by inactivated MEK–MAPK cascades downstream of fibroblast growth factor receptor 1 (FGFR1) and inhibit the apoptosis checkpoint molecule BCL2, being degraded via the proteasome. Then, inactivated BCL2L1/BCL-XL allows BOX, BAX, and Bcl-2 homologous antagonist/killer (BAK1) activation, responding to ER stress through the IRE1α-XBP1 UPR pathway. The activated BAX/BAK1 promotes the release of CYC1 (cytochrome c 1) from mitochondria, which would form apoptosomes together with CASP9 (caspase 9) and CASP3 (caspase 3).

### GeneMANIA-based upstream analysis

**Figure 4:**
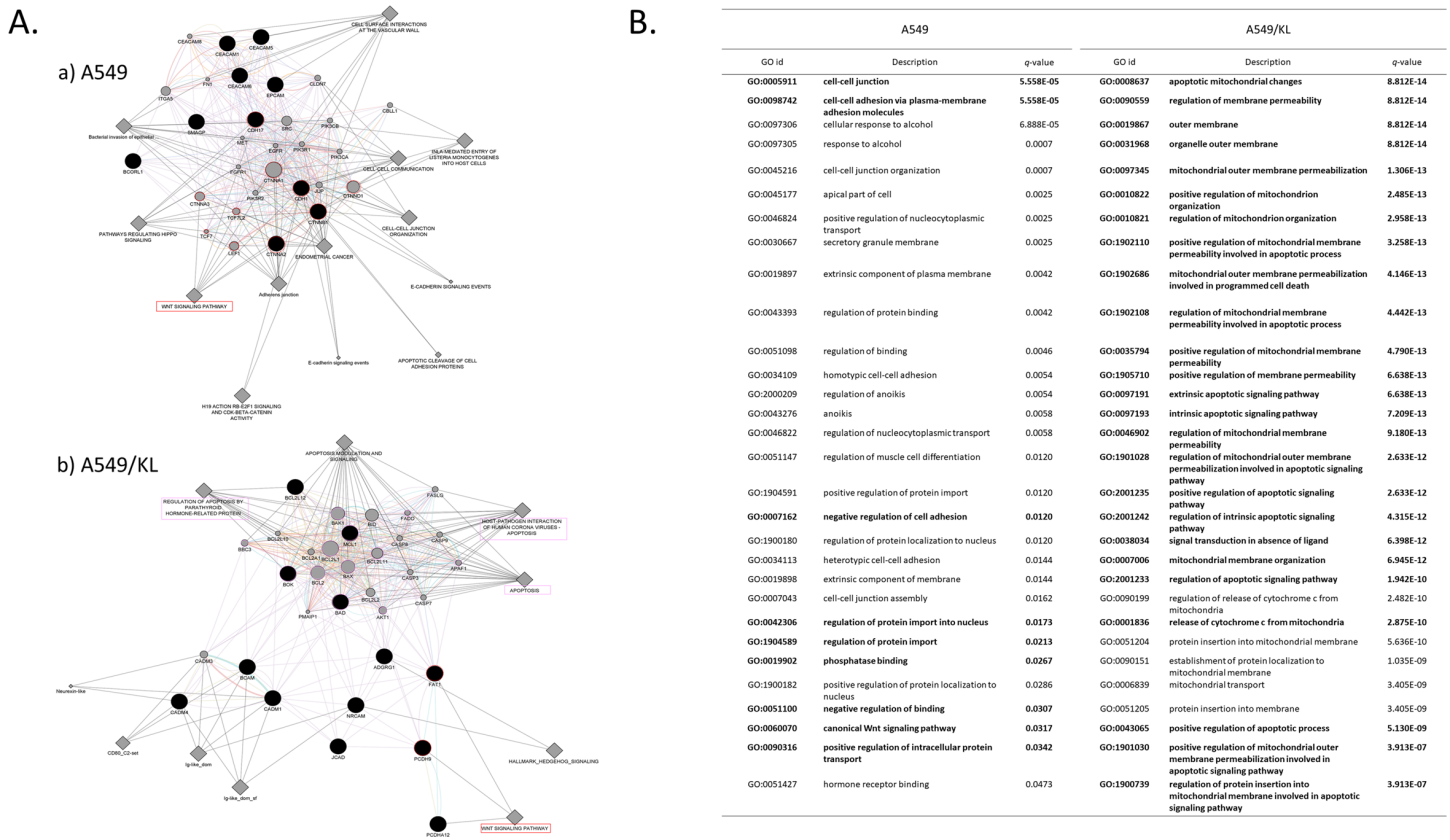
(**A**) The GeneMANIA analysis of the 23 adhesion and apoptosis-related proteins unregulated in the (**a**) A549 and (**b**) A549/KL cells, each other. Red circles denote proteins associated with the Wnt signaling pathway, and pink circles are associated with the apoptosis pathway. (**B**) Top thirty annotations on GO biological processes obtained by GeneMANIA for the A549 and A549/KL cells (*q-*values denote the Benjamini–Hochberg FDR multiple testing correction).

Among the total proteins identified, we focused on the 23 adhesion- and apoptosis-related proteins upregulated between A549 and A549/KL (Table 2). GeneMANIA upstream analysis (University of Toronto, Ontario, Canada: http://genemania.org/)^17^ via Cytoscape app (version 3.9.1) (https://cytoscape.org/)^18^ was applied to those proteins to find an additional 20 interacting genes/proteins (Fig. 4). Proteins upregulated in the A549 or A549/KL cells are shown as large black-filled circles, and their upstream and/or interacting molecules that were found are shown as gray-filled circles, which are ordered by relatedness to the query genes/proteins in bipartite layouts (Fig. 4A)^17^.

Regarding the A549 cells, the 10 adhesion- and apoptosis-related proteins upregulated include CDH1 (E-cadherin), epithelial cell adhesion molecule (EpCAM), CTNNB1 (β-catenin), BCL-6 corepressor-like protein 1 (BCORL1), and carcinoembryonic antigen (CEACAM5) and were found to interact with catenin α-1, SRC, EGFR, MET, LEF1 (TCF1-α), and FGFR1. The biological process (GO) annotated to the molecular networks found that A549 cells included extrinsic components of membranes, cell–cell junction, cell–cell adhesion via plasma–membrane adhesion molecules, negative regulation of cell adhesion, and negative regulation of binding (Fig. 4B). Their related networks included E-cadherin signaling events, the Wnt signaling pathway, and pathways regulating HIPPO signaling. Characteristically, the key molecules of the Wint/β-catenin signaling pathway included β-catenin, catenin α-1, E-cadherin, TCF1-α, and TCF7L2 (T-cell factors/lymphoid enhancer factors), CTNNA3 (α-T-catenin), CTNNA2 (α-N-catenin), and CDH17 (Cadherin-17) (Fig. 4Aa). β-catenin has the leading role in the Wnt signaling pathway associated with EMT in cancer progression. Upregulation of N-cadherin (CDH2) followed by downregulation of E-cadherin, which is referred to as the “cadherin switch,” is the hallmark of EMT, by which β-catenin activates several key signaling pathways, such as Wnt/β-catenin and the lymphoid enhancer factor (LEF)/T-cell factor (TCF), leading to the acquisition of invasive and metastatic potential. Canonical Wnt signaling leads to the accumulation of β-catenin in the cell membrane, and β-catenin unphosphorylated is stabilized in the cytoplasm, followed by its translocation into the nucleus to activate the transcription of *TCF/LEF*-target genes, key factors in cell proliferation and invasion, including FN1 and c-Myc^22,23^. Cadherin-17 belongs to the Wnt/β-catenin signaling pathway, and it was shown that targeting the cadherin-17 gene (*CDH17*) by RNA interference-mediated knockdown inhibited the proliferation of both primary and highly metastatic hepatocellular-carcinoma (HCC) cell lines in vitro and in vivo^24^.

**Figure 5:**
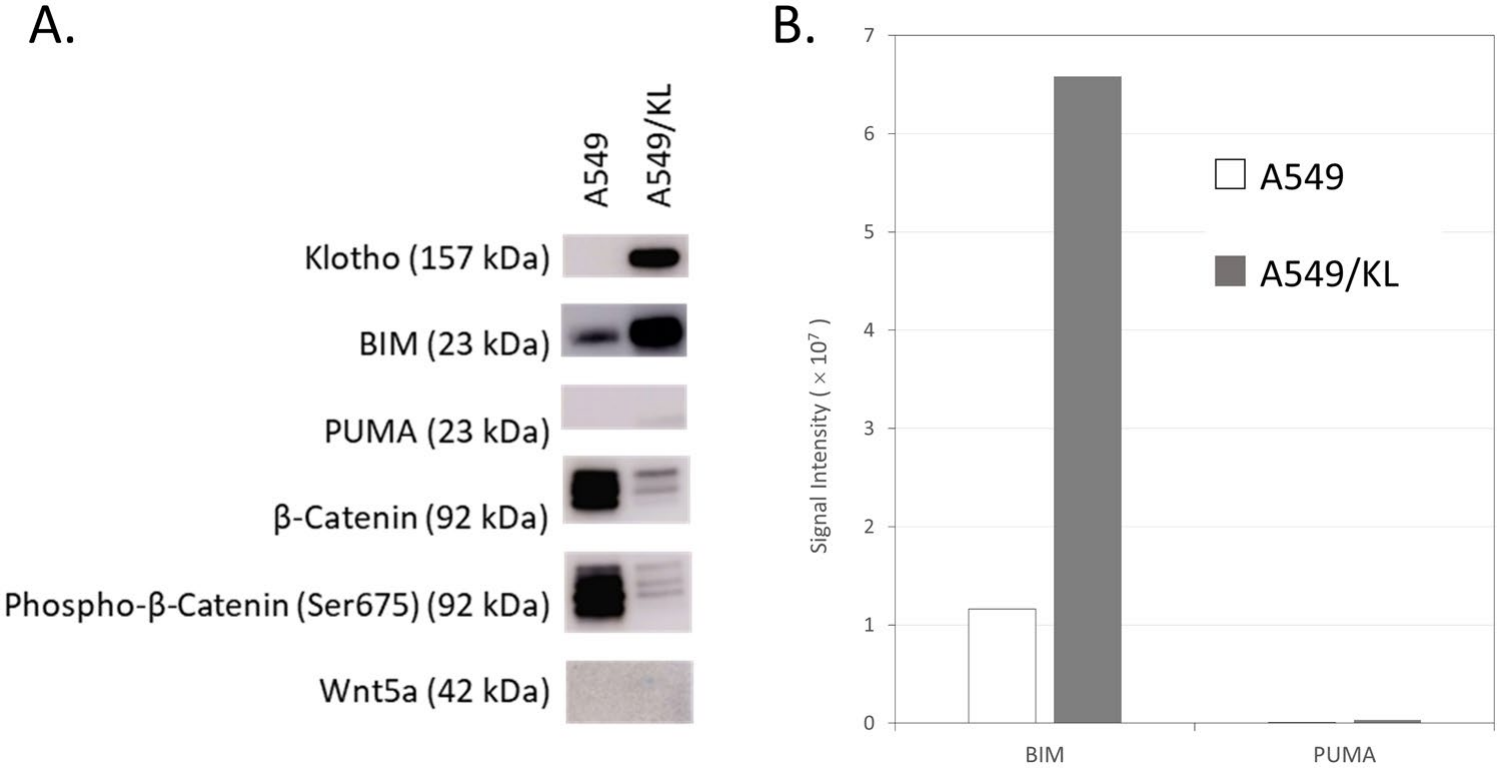
(**A**) Western blot analysis for A549 cells and A549/KL cells using anti-Klotho, anti-Bim, anti-Puma, anti-β-catenin, anti-Phospho-β-Catenin, and anti-Wnt5a antibodies. Wnt5a is a marker of non-canonical Wnt-Frizzled pathway. All gel images are presented in Figure S6 as full as possible length gels and blots with membrane edges visible. (**B**) The graph shows the relative expression levels of Bim and Puma in the A549 and A549/KL cells quantified by ImageQuant TL ver.8.1 (Cytiva) and demonstrates highly upregulated Bim levels in the A549/KL cells, whereas Puma was detected only at a background level.

Our proteomic analysis could not capture N-cadherin but could capture E-cadherin, as Table 2 and Fig. 4Aa show, suggesting that our A549 strain did not acquire a highly enhanced but rather a limited mesenchymal characteristic for invasion and/or metastasis. The extent of the “cadherin switch” seems to depend on a balance between unstable phosphorylated β-catenin and stable unphosphorylated β-catenin in the cytoplasm. The former leads to proteasomal degradation and the latter is translocated into the nucleus. N-cadherin binds to β-catenin, and the expression levels of N-cadherin seem to depend on the balance of β-catenin accumulated between the cell surface and the cytoplasm, where Wnt/β-catenin signaling functions as a rheostat^23^. Nevertheless, our GeneMANIA analysis results of upregulated proteins associated with the A549 strain (Fig. 4Aa) implicated the activation of canonical Wnt/β-catenin signaling. The main proteins participating in the Wint/β-catenin^25^ and the Wnt signaling pathways, together with matrix metalloproteinases, from our proteome data are listed in Table 2C and D.

On the other hand, the 13 adhesion- and apoptosis-related proteins upregulated in the A549/KL cells include BCAM, neuronal cell adhesion molecule (NRCAM), protocadherin 9 (PCDH9), protocadherin Fat1 (FAT1), BOK, Bcl-xL/Bcl-2–associated death promoter (BAD), and induced myeloid leukemia cell differentiation protein Mcl-1 (MCL1). Interestingly, their interacting molecules were apoptosis checkpoint molecules, including Bcl2-interacting mediator of cell death (BCL2L11, Bim), p53 upregulated modulator of apoptosis (BBC3, Puma), BAK1, BAX, BCL2, BCL2L1, B-cell lymphoma-extra large (Bcl-XL or BCL-XL), and BH3-interacting domain death agonist (BID). Other apoptosis-related molecules were FAS-associated death domain protein (FADD), apoptosis-mediating surface antigen FAS (FASLG), apoptotic protease activating factor-1 (APAF1), PMA-induced protein 1 (PMAIP1), AKT1, CASP3, CASP7, CASP8, and CASP9 (Fig. 4Ab).

The results obtained above are consistent with those from IPA-based upstream and causal network analysis, supporting that both Bim and Puma are most likely master regulators of molecular networks affected by the *KL* gene. Finally, our western blot analysis confirmed Bim as a possible master regulator in molecular networks altered by the *KL* gene transfection to the A549 cells (Fig. 5).

A web-based survival analysis (KMplot) for mRNA data of lung carcinoma indicated that better overall survival was associated with the upregulated levels of Bim, BOK, and MCL1 than with BLC2L1 (BCL2-XL) and N-cadherin (Figure S5)^26^.

Several key molecules relevant to the Wnt signaling pathway were also found, which include PCDH9 and FAT1. PCDH9 inhibits EMT and cell migration^27^, and FAT1 is known as a tumor suppressor, whereas it is frequently mutated, and inhibits Wnt/β-catenin signaling by negatively regulating β-catenin nuclear translocation and its transcriptional activity^28,29^. The biological processes (GO) dominantly annotated were the extrinsic and intrinsic apoptosis signaling pathway, apoptotic mitochondrial changes, positive regulation of mitochondrial membrane permeability involved in the apoptotic process, the release of cytochrome c from mitochondria, and positive regulation of mitochondrial outer membrane permeabilization involved in the apoptotic signaling pathway (Fig. 4B).

Activation of Bim was reported to suppress cancer metastasis by inhibiting MCL1^30,31^ and EMT-related N-cadherin expression^11^. Conversely, it has been shown that upregulation of N-cadherin suppresses Bim expression^32^. Our study has not provided evidence that Klotho directly interacts with N-cadherin or directly perturbs the Wnt/β-catenin signaling and apoptosis signaling via Bim. However, it was suggested that the extracellular domain of Klotho binds to several Wnt ligands, which inhibit the potential ability to activate Wnt signaling^33^. Klotho is the co-receptor of FGFR1, which is the specific receptor for the phosphaturic hormone fibroblast growth factor-23 (FGF23). There are two types of Klotho proteins: soluble Klotho (sKlotho) and secreted Klotho. The sKlotho protein binds to multiple ligands of Wnt and suppresses various gene transcriptions. Upregulated sKlotho has previously been demonstrated to attenuate renal fibrosis by suppressing Wnt signaling in mice models^34,35^. Accumulating data have suggested crosstalk between Wnt/β-catenin signaling and regulation of Klotho and FGF23^36^.

In this study, the Klotho protein and various key proteins associated with cancer-, apoptosis-related, and Wnt/β-catenin pathways were quantitively identified (Table 2). A high expression level of the Klotho protein was observed in the A549/KL cells, confirming its successful establishment of the A549/KL strain, whereas the detected upregulation of the EMT markers VIM and ZEB1 might reflect a complicated trait of the A549/KL strain, but this remains unclear.

Our MS-based proteomic analysis captured several apoptosis-related proteins, including BAK1, BAX, Bax inhibitor 1 (BI1), BCL2L1 (BCL-XL), APAF1, FADD, and death receptors DR4/5. Upregulation of BAK1 and downregulation of BAX, BI1, and BCL-XL were observed in A549/KL. Upregulated expression of the death receptor 4 TNF-related apoptosis-inducing ligand-receptor 1 (TRAILR1) and death receptor 5 (DR5) TRAILR2 was observed in A549/KL, which might be attributed to release from the selective inhibition by EMT^37^.

Regarding the A549/KL cells, we identified the co-receptor of Wnt ligands, slightly upregulated Frizzled-2/5/6/7 and low-density lipoprotein receptor-related protein 5/6 (LRP5/6), and slightly upregulated GSK3β. Those proteins form the signalosome complex, in which LRP5/6 are phosphorylated when the Wnt signaling is activated (Wnt ON). Proto-oncogene Src directly phosphorylates LPR6 and Frizzled, which was observed to be downregulated in A549/KL. Notably, the phosphorylation of a protein/peptide generally reduces its electrospray ionization efficiency in MS. The above observations likely reflect inactivated Wnt signaling (Wnt OFF) in A549/KL. Wnts were not detected, but Wnt ligand secretion mediator, also known as Wntless (WLS), which is required for the secretion of all Wnts, was detected in both traits^38^.

Moreover, upregulation of fibroblast growth factor 2 (FGF2), FGFR1, FGFR4, and EGFR were also found in A549/KL (Table 2B). The binding of FGF2 to FGFR1, the most important FGF2 receptor, activates downstream signaling, including MAPK/ERK, PLCg, and PI3K/AKT pathways^39^. Upregulation of FGFR1 and FGF2 in the A549/KL cells might suggest that FGF2-activated FGFR1 signaling induces FGFR1 internalization, thereby stabilizing FGFR1 expression^40^. Upregulation of FGFR1 in NSCLC cell lines by hypoxia induces subsequent activation of MAPK cascades, leading to attenuated induction of the pro-apoptotic factor Bim, which drives acquired resistance to EGFR tyrosine kinase inhibitors^41–44^. Therefore, combining EGFR TKIs with FGFR1 inhibitors or MEK inhibitors is considered an attractive therapeutic strategy for NSCLCs^41^.

## Discussion

The results of our invasive assay and lung tumor-bearing mice model confirmed that Klotho suppresses invasive and metastatic potential, in which N-cadherin suppression is assumed to be induced by the *KL* gene^11^. We conducted in-depth MS-based quantitative proteomic analysis to elucidate protein expression profiles of A549 and A549/KL cells. The proteomic data successfully applied to WGCNA analysis identified 60 data-driven WGCNA co-expression modules associated with the A549 and A549/KL cells. Additionally, the two-step WGCNA analysis of the obtained MS-based proteomic data identified one WGCNA module, the WM55-2 (turquoise-2) consisting of 582 member proteins as most significantly associated, by far, with the A549/KL cells. Both the upstream regulator and causal network analysis by IPA for the turquoise-2 module and GeneMANIA analysis applied to the adhesion- and apoptosis-related proteins upregulated in the A549/KL cells implicated Bim and/or Puma as the most likely master regulators underlying molecular networks affected by Klotho occurring in the A549/KL cells. Our western blotting analysis confirmed Bim as a significant regulator (Fig. 5).

Involvement of canonical Wnt signaling is most likely responsible for the invasive and metastatic potential of the A549 trait, and was inactivated in the A549/KL cells, which might be evidenced by the expressions of PCDH9 and Fat1. Loss of N-cadherin could be explained partly by the “cadherin switch” regulated centrally by β-catenin in the context of inactivated Wnt signaling. The GeneMANIA analysis results implicated the involvement of two different signaling axes: FGF-FGFR and Wnt/β-catenin. Interestingly, our quantitative proteomic data exhibited upregulated expression of the FGFR1 protein in A549/KL cells. A speculative scenario is that Klotho suppresses Wnt/β-catenin signaling and abolishes phosphorylation downstream of MEK/ERK in the FGF-FGFR signaling, which prevents phosphorylation of Bim and synergically Wnt co-receptor LRP6^40,44^.

**Figure 6:**
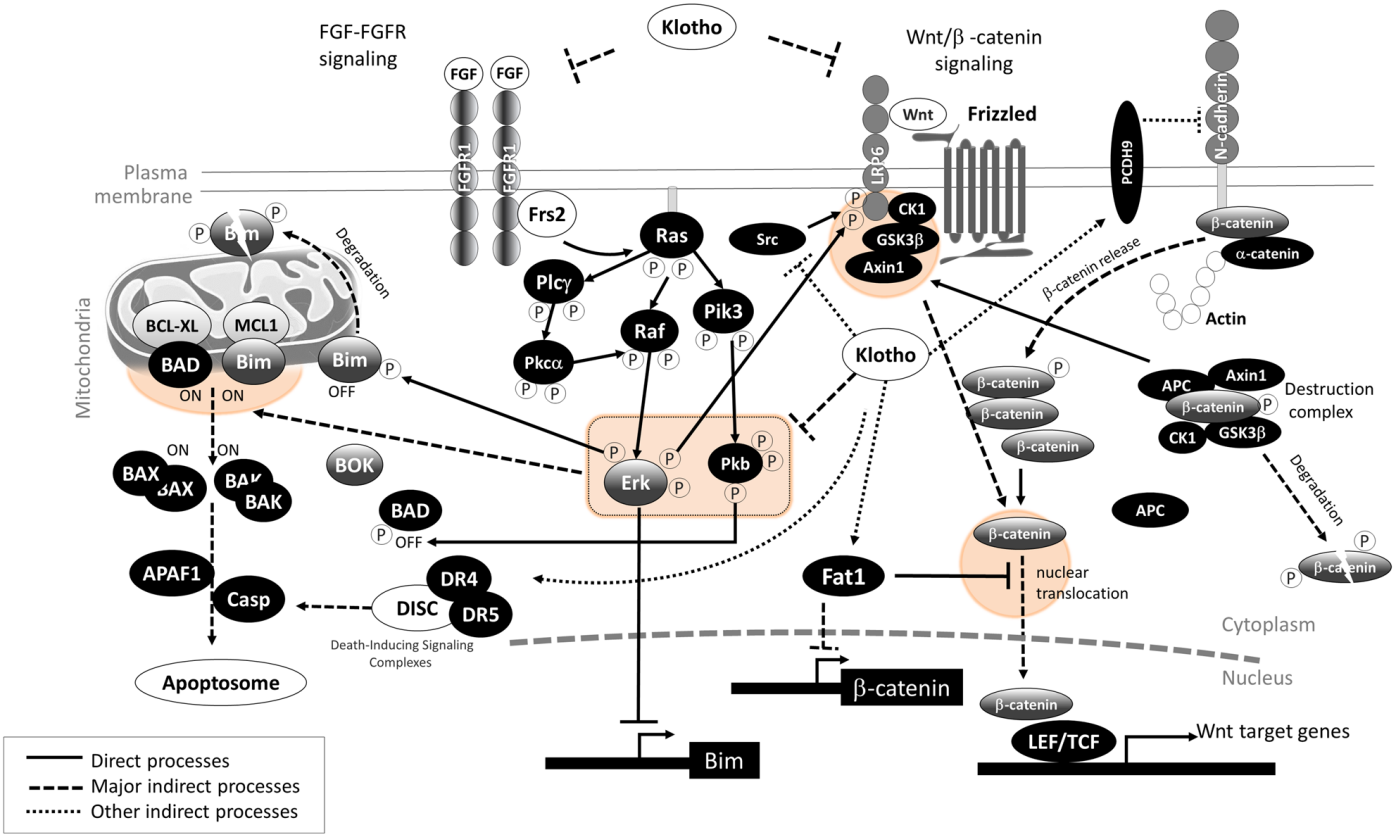
A model of the cross-talk between FGF-FGFR and Wnt/β-catenin signaling in the A549 cells, with which Klotho negatively interacts in the A549/KL cells. When Wnt/β-catenin signaling is activated (Wnt ON), Wnts bind to the Wnt coreceptors Frizzled receptors (FZDs) and LRP5/6, followed by phosphorylation of LRP5/6, and induce the clustering of Wnt/Frizzled/LRP6 into the signalosome complexes, which allows for Axin and GSK3β binding to them. The signalosomes amplify the Wnt signal, where phosphorylated LRP5/6 triggers the successive recruitment of Axin1/GSK3β/β-catenin to the cytoplasmic tail of LRP5/6, resulting in the displacement of GSK3β from the Axin1/β-catenin complex, inhibition of GSK3β and decreased phosphorylation of β-catenin. Thus, β-catenin is stabilized and accumulated as free β-catenin in the cytosol, which is subsequently translocated into the nucleus and activates the TCF (T-cell factor) /LEF (lymphoid enhancer factor)-dependent transcription of Wnt target genes. N-cadherins are linked to the actin cytoskeleton participating in the adherens junction, through their binding to α-catenins and β-catenins. The release of β-catenin from adherens junctions is induced by oscillatory fluid flow, and Wnt/β-catenin signaling competes for the same cellular pool of β-catenin. Activated FGF-FGFR signaling turns on downstream signaling such as the RAF–MEK–ERK and PI3K–PKB pathways. Both pathways promote cell survival by antagonizing pro-death proteins, Bim and BAD. ERK directly phosphorylates Bim, followed by its proteasomal degradation^47^, and also PKB can directly phosphorylate BAD^48^. Both the ERK and PKB also promote the expression of pro-survival proteins BCL-XL and MCL1^49,50^. Klotho interacts negatively with the Wnt/β-catenin signaling (Wnt OFF), which results in the phosphorylation of cytoplasmic β-catenins followed by their rapid degradation, mediated by the destruction complex, and also induces Protocadherin 9 and Fat1, suppressing EMT and Wnt/β-catenin signaling by negatively regulating β-catenin nuclear translocation and its transcriptional activity. Klotho suppresses the downstream of FGFR signaling, ERK, and PKB, by which activated Bim and BAD promote pro-apoptosis signalings, through activated BAX/BAK, APAF1, and Caspases, and synergically which prevents phosphorylation of Wnt co-receptor LRP6. Moreover, Klotho induces activation of DR4/5 death receptor signaling.

Klotho appears to function as an antagonist of the Wnt signaling pathway. It can inhibit the activation of this pathway. The canonical Wnt pathway involves the stabilization and nuclear translocation of β-catenin, which leads to the expression of specific target genes associated with cell proliferation and survival. Klotho, especially the secreted form known as sKL, has been shown to reduce the active form of β-catenin (non-phosphorylated or dephosphorylated on specific residues) and decrease the expression of Wnt target genes such as c-Myc and Cyclin D1^45^. It was also suggested that Klotho acts as a tumor suppressor and an inhibitor of the Wnt/β-catenin pathway in HCC^46^. A simple diagram summarizing the results of this study together with the literature regarding Klotho, BIM, and Wnt/β-catenin signaling^47–50^ is presented in Fig. 6.

A limitation of this study is the small sample size using only the A549 cell line. Further study is required to validate our results and identify which molecules targeted by Klotho are responsible for the A549/KL trait.

In conclusion, we successfully applied in-depth MS-based proteomics to identify and quantify proteins expressed in A549 and A549/KL cells. Upstream analyses using both WGCNA and GeneMANIA were successfully applied to their proteomic datasets and identified the functional protein networks significantly associated with both cell traits.

## Materials and methods

### Cell culture and transfection

A549, a human lung adenocarcinoma cell line was maintained in Dulbecco’s modified Eagle’s medium (DMEM; cat. no. D6429; Millipore Sigma) supplemented with 0.1% sodium bicarbonate, L-glutamine, sodium pyruvate, 10% heat-inactivated fetal bovine serum (FBS; cat. no. F2442; Millipore Sigma), and penicillin (100 U/mL) in a humidified atmosphere of 5% CO_2_ at 37 °C. The Lipofectamine 3000® transfection reagent (Invitrogen; Thermo Fisher Scientific Inc.) was used to transfect GFP–klotho plasmids A549 cells according to the manufacturer’s instructions. The transfection reagent was used to transfect 0.25 –1 × 10^6^ cells with 2 µg of plasmid DNA, which were then incubated for 2 h in a humidified atmosphere of 5% CO_2_ at 37 °C. The cells were washed, and then medium that included FBS was added. The GFP–*Klotho* plasmid, which was previously provided by Dr. Nabeshima (Foundation for Biomedical Research and Innovation, Kobe, Japan), was transfected into the A549 cells, and 24 h later the cells were pelleted (almost 1 mg) by centrifugation at 1,500 rpm for 10 min and resuspended in phosphate-buffered saline (PBS) to a final density of ~ 2.9 × 10^6^ cells/mL, and the suspension was then filtered through Cell Strainer (40 μm) to remove cell aggregates. A FACSCanto II (BD Biosciences) with the activation set at 488 nm and fluorescence-emission monitoring at 508 nm (GFP), was used to sort the GFP-positive cells. FlowJoTM v.10.7 software (TreeStar Inc.) was used to perform the data acquisition and analysis. At least 10,000 events were collected for each analysis. The forward-scatter and side-scatter parameters were used to eliminate the dead cells and debris, and the remaining cells were sorted into GFP-positive and GFP-negative populations ^11^.

### Isolation of clones expressing Klotho

Following GFP–*Klotho* transfection, the GFP-positive cells were cultured with 0.1% sodium bicarbonate, L-glutamine, sodium pyruvate, and 10% FBS, penicillin (100 U/mL) in a humidified atmosphere of 5% CO_2_ at 37 °C for 2–3 weeks, and the colonies were then harvested. A limiting dilution method was used to obtain a single clone. Western blotting was performed to check several colonies for Klotho expression, and stably overexpressing Klotho cell line, A549/KL, was established^11^.

### Cell invasion assay

A CytoSelect 24-well cell invasion assay kit (Cell BioLabs, Inc.) was used to perform cell invasion assays.This assay kit contains polycarbonate membrane inserts (8 μm pore size). The upper surface of the insert membrane is coated with a uniform layer of dried basement membrane matrix solution. The basement membrane layer serves as a barrier to discriminate invasive cells from non-invasive cells. Invasive cells can degrade the matrix proteins in the layer and ultimately pass through the pores of the polycarbonate membrane. Finally, these cells are dissociated from the membrane and subsequently detected by CyQuant® GR Dye (Invitrogen).

A cell suspension was placed in an upper chamber in serum-free media. A549 and A549/KL cell suspensions containing 3.0 × 10^5^ cells were seeded in the chamber, and each cell was seeded at three different locations. After incubating for 24 − 48 h at 37 °C in a 5% CO_2_ atmosphere, the invasive cells were dissociated from the membrane by adding Cell Detachment Buffer to the lower chamber. Invasive cells were lysed by adding Lysis Buffer with CyQuant GR Fluorescent Dye. Subsequently, each sample was transferred to a 96‐well microtiter plate and quantified by reading the fluorescence at 480 nm/520 nm with a plate reader.

### Mouse lung metastasis model

Female 5-week-old BALB/cSlc-nu/nu mice were obtained from MediRidge Company, Limited (Tokyo, Japan). All animal experiments were conducted according to protocols approved by the Animal Care and Use Committee of Nippon Medical School (approval number: 2021–030). A549 and A549/KL cells (5.0 × 10^6^ cells) in 100 μL PBS were injected into the tail veins of mice (three mice/group) to generate lung tumor metastases. The mice injected with cells were sacrificed at 8 weeks. Excised mouse lungs were fixed in formalin and embedded in paraffin. Tumor metastasis to the lungs was assessed by hematoxylin and eosin (H&E) staining. Fifteen sections of lung tissue from three mice, five sections of lung tissue per mouse, were evaluated.

### Sample preparation for LC–MS/MS

#### Reagents and materials

The protease inhibitor (Protease Inhibitor Cocktail Tablets, complete, Mini, EDTA-free Tablets) was purchased from Roche Diagnostics (Indianapolis, IN, USA). Benzonase (Benzonase® endonuclease, purity grade I (≥ 99%) suitable for biopharmaceutical production) was purchased from Merck (Whitehouse Station, NJ, USA). Ammonium bicarbonate (AMBIC), PBS, dithiothreitol (DTT), iodoacetamide (IAA), and triethylammonium bicarbonate (TEAB) were purchased from Sigma (St. Louis, MO, USA). Sequencing-grade trypsin was purchased from Promega (Madison, WI, USA). BCA reagent was purchased from Thermo Fisher Scientific (Pierce Biotechnology, Rockford IL, USA). Acetonitrile, trifluoroacetic acid, formic acid, and methanol were purchased from Kanto Chemical Co. Ltd. (Tokyo, Japan). Sodium dodecyl sulfate (SDS) was purchased from Amersham Biosciences (Amersham, UK). Phosphoric acid was purchased from NACALAI TESQUE, INC. (Kyoto, Japan). All water used in this study was Milli-Q ultrapure water (Merck Millipore, Billerica, Massachusetts, USA). All reagents were of analytical grade.

#### Sample preparations

The samples analyzed in this experiment consisted of A549 adherent cells and A549 cells transfected with the *KL* gene. For cell culturing, 75 cm^2^ culture flasks were used with w DMEM + 10% FBS medium added. Cells at 80% confluency were collected by the following procedure. First, the old medium was removed, and the cells were washed with 10 mL of PBS (–). Then, 2 mL PBS (–) was added, and the cells were scraped off with a cell scraper. The cell suspension was centrifuged at 1500 rpm for 3 min, and the pellet was resuspended in 1 mL PBS (–) and transferred to a 1.5 mL tube. The tube was centrifuged at 700 g for 10 min, and the collected cell pellet was stored at − 80 °C.

A 50-µL aliquot of lysis buffer was added to the harvested cell pellet sample. The component of the lysis buffer was as follows: protease inhibitor (prepared at a 14-fold dilution) and benzonase were added to 10% SDS in 10 mM TEAB, pH 7.55. A Pierce™ BCA Protein Assay Kit (Thermo) was used to quantify the dissolved protein sample solution to determine the protein concentration. All samples were prepared to have a total starting material of 100 μg protein.

The samples, A549 adherent cells, and A549 cells transfected with the *KL* gene were divided into three portions, resulting in six samples that were subjected to sample preparation using S-trap™^51–55^. For each of the six samples, DTT and IAA were used for reduction and alkylation, followed by bringing the samples back to room temperature and adding phosphoric acid to a final concentration of 1.2%. Then, S-trap buffer (100 mM TEAB in 90% methanol, pH 7.1) was added in a volume sixfold higher than that of the sample, and the mixture was applied to the S-trap micro spin column. The protein retained on the S-trap was washed five times with S-trap buffer. Then, a 1-μg equivalent of trypsin was added, and the samples were incubated at 47 °C for 1 h, then incubated overnight at 37 °C.

The samples were then eluted using the following method. First, 40 μL of 50 mM TEAB was added to the S-trap micro and centrifuged at 4000 g for 1 min. Then, 40 μL of 0.2% formic acid was added to the S-trap micro and centrifuged at 4,000 g for 1 min. Finally, 35 μL of 0.2% formic acid in 50% acetonitrile was added to the S-trap micro and centrifuged at 4,000 g for 1 min. The entire flow-through was collected, and the solvent was completely evaporated on a SpeedVac evaporator. The sample was then reconstituted in 20 μL of 0.1% trifluoroacetic acid in 2% acetonitrile for LC–MS/MS analysis.

### Proteomic analysis by liquid chromatography–tandem mass spectrometry (LC–MS/MS)

The eluted samples were separated by nanoflow reversed‐phase LC followed by analysis on a Q-Exactive mass spectrometer (Thermo Fisher Scientific, San Jose, CA) equipped with a Dream spray nano-electrospray ionization source (Dream spray, AMR Inc., Tokyo, Japan). The LC instrument was an Ultimate 3000 dual‐solvent delivery system (Thermo Fisher Scientific) equipped with a PAL LSI auto‐sampler (CTC Analytics AG, Zwingen, Switzerland). The samples were loaded onto a capillary reversed-phase separation column packed with 1.6-μm-diameter gel particles with a 120 Å pore size (AURORA C18, 250 × 0.075 mm, IonOpticks). Eluent A was 0.1% formic acid, and eluent B was 100% acetonitrile. The column was eluted at a flow rate of 0.2 μL/min with a concentration gradient of A + 5% B to 35% B over 100 min and from 35% B to 95% B over 1 min, with subsequent isocratic elution at 95% B for 8 min and then a return to initial conditions from 95% B to 5% B over 1 min for re-equilibration.

The mass spectrometer was operated in DIA mode in which the MS acquisition with a mass range of m/z 380–1600 was automatically switched to MS^2^ acquisition under the automated control of Xcalibur software 3.1 (Thermo Fisher Scientific). DIA was performed with staggered isolation windows, a loop count of 25.0 m/z, and a normalized collision energy of 27 with a 200-ms maximum injection time at 70,000 resolution. In the DIA mode, each cycle consisted of an MS^1^ scan of 380–1600 m/z with 70,000 resolution and an AGC target of 1 × 10^6^, followed by 48 MS^2^ scans of 400–1600 m/z with a resolution of 35,000 and an AGC target of 1 × 10^6^ ^56^.

### Protein identification

Acquired raw data were processed by DIA by Neural Networks (DIA-NN) for proteomics analysis. DIA-NN is known to be particularly useful for high-throughput proteomics applications because it improves the performance of protein identification and quantification in traditional DIA mode proteomics applications, enabling fast and reliable protein identification^13,57^.

The algorithm for DIA-NN is described as follows. DIA-NN version 1.8.1 in library-free mode was used with the same Uniprot FASTA database. The Protein Database was applied to the human database (UniProt Reference Proteome—Homo sapiens, Taxonomy 9606—Proteome ID UP000005640 -. 20,373 entries—UniProt release 2022_03, reviewed human canonical)^13^.

Precursors of charge state 1–4, peptide lengths 7–30, and peptide m/z-values from 300 to 1800 were considered with a maximum of one missed cleavage. A maximum of one variable modification per peptide was considered. We used cysteine carbamidomethylation as a fixed modification, N-terminal methionine excision as a variable modification, methionine oxidation as a variable modification, and N-terminal acetylation as a variable modification. Precursor False Discovery Rate (FDR) was then filtered at 1%^13,57^.

### Weighted correlation network analysis (WGCNA)

The similarity in protein expression patterns for all protein pairs was calculated according to their pairwise Pearson’s correlation coefficient *r* (i.e., the similarity between proteins i and j was defined as (1–*r*_i,j_)/2, where *r*_i,j_ is the Pearson’s correlation coefficient of the protein expression pattern between the two proteins). We performed a network topology analysis using the adjacency of an unsigned network, *a*_ij_ =|cor(*x*_*i*_, *x*_*j*_)|^β^ between gene expressions x(i) and x(j), for various soft-thresholding powers ranging from β = 1 to 20 to choose an optimal value of balance between independence and mean connectivity, where A topological overlap matrix (TOM) that considers topological similarities between a pair of proteins in the network was then generated from the resultant scale-free co-expression network. We used dissimilarity according to TOM (1 − TOM) to generate a tree by hierarchical clustering, and dynamic tree-cutting to trim the branches to determine protein modules^16^.

The modules were summarized by the first principal component, which is referred to as eigen proteins in the text because they express the highest connectivity in the module. Module membership, defined as the correlation between the protein expression profile and the module eigen-protein, was measured with values ranging from 0 to 1, with “0” representing a gene that is not part of the module and “1” representing high connectivity with the module. Subsequently, the module-trait association was determined by the correlation between the module eigen-protein and the traits A549 and A549/KL. A protein module was summarized by the top hub protein (referred to as the “eigen-protein”) with the highest connectivity in the module. The two-step WGCNA analyses were performed using the WGCNA R-package^14^ implemented in RStudio.

### Protein–protein interaction (PPI) network construction

We used the STRING database (version 11.5) to construct a PPI network for a protein module (https://string-db.org/)^18^. STRING networks were calculated under the criteria for linkage with experiments, databases, text mining, and co-expression using the default settings (medium confidence score: 0.400; network depth: 0 interactions). Functional enrichment results were obtained for canonical pathways with a *p*-value < 0.05. Protein networks were subsequently exported to Cytoscape (version 3.9.1) (https://cytoscape.org/) from the STRING database^18^. The hub proteins in each module were identified according to their intramodular connectivity and their correlation with module eigenproteins. The proteins inside the co-expression modules exhibit high connectivity and the proteins within the same module may have similar roles. The top 10 high-degree proteins were identified using the *cytoHubba* plugin^19^. The top-ranked proteins in each module were considered hub proteins and designated “highly connected proteins.” Functional enrichment results were obtained for canonical pathways by considering a network bias-corrected *p* value of < 0.05 for statistical significance.

Quantile normalization of protein expression data obtained by MS-based proteomic analysis conducted in DIA mode and the pairwise correlation of identified WGCNA modules were performed in JMP software (SAS Institute, Cary, NC, USA). The Intervene Shiny App was used to visualize pairwise correlation (https://intervene.shinyapps.io/intervene/)^58^.

### Ingenuity pathway analysis (IPA)

IPA software was used to predict upstream regulators, causal networks, and canonical pathways^16^. Quantile-normalized protein expression data of the selected modules were used as input datasets. Both the upstream regulators and causal networks (*p* < 0.05) predicted from the WGCNA network modules were significantly associated with the A549 or A549/KL trait in which the activation and inhibition of a predicted network were defined by *z*-values that were > 2.0 and <  − 2.0, respectively. The upregulation was defined by *z*-values > 1.5 and < 2.0, whereas downregulation was defined by *z*-values >  − 2.0 and <  − 1.5.

### GeneMANIA

The list of proteins in gene name/accession number was submitted to the GeneMANIA (http://genemania.org/)^17^ via Cytoscape app (version 3.9.1) (https://cytoscape.org/), where an additional 20 related genes/proteins were searched with all the interaction networks consisting of co-expression, co-localization, genetic interactions, pathways, physical interactions, predicted, and shared protein domains with 20 attributes using GO biological-based weighting. Large black-filled circles are query genes/proteins, and their upstream and/or interacting molecules were shown in gray-filled circles, which are ordered by relatedness to the ten query genes/proteins in bipartite layouts. A colored relationship line corresponds to the respective interaction network category.

### Western blot

Overnight incubation was performed at 4 ℃ with rabbit primary antibodies against BIM (#2933, 1:1,000; Cell Signaling Technology) and PUMA (#4976; 1:1,000; Cell Signaling Technology). After washing with Tris-buffered saline and 0.1% polysorbate 20, membranes were incubated with horseradish peroxidase–conjugated secondary anti-rabbit IgG antibodies (#7074; 1:1,000; Cell Signaling Technology) at room temp for 1 h. 1 h incubation was performed at RT with rabbit primary antibodies against β-catenin (#8480, 1:1,000; Cell Signaling Technology), Phospho-β-Catenin (Ser675) (#4176, 1:1,000; Cell Signaling Technology), Wnt5a (ab235966, 1:1,000; Abcam). Regarding Klotho-GFP, 1 h incubation was performed at RT with Rat primary antibodies against Klotho (KO603, 1:1,000; Medicinal Chemistry Pharmaceutical Co.) and horseradish peroxidase–conjugated secondary anti-Rat IgG antibodies (5220–0365; 1:20,000; SeraCare). Immunoreactive protein was detected with enhanced chemiluminescence substrate, and band intensities were quantified by ImageQuant TL ver.8.1 (Cytiva). After visualization of the target protein, membranes were stripped and reincubated with antibodies against β-actin (#4967; Cell Signaling Technology)^11^.

### Supplementary Information

Supplementary Information.

## Data Availability

The unfiltered MS datasets generated and analyzed in this study have been deposited in ProteomeXchange (http://proteomecentral.proteomexchange.org) and jPOST, with the dataset identifiers PXD042978 and JPST002200, respectively.
