# Supplementary material for: Protein interaction networks characterizing the A549 cells Klotho transfected are associated with activated pro-apoptotic Bim and suppressed Wnt/β-catenin signaling pathway
Source: Sci Rep. 2024 Jan 25;14:2130. doi: 10.1038/s41598-024-52616-0 (PMC10808115; doi:10.1038/s41598-024-52616-0)
Supplement: Supplementary file 1 — Supplementary Information. [file 41598_2024_52616_MOESM1_ESM.pdf]

# Supplementary Information File 1

## Protein interaction networks characterizing the A549 cells Klotho transfected are associated with activated pro-apoptotic Bim and suppressed Wnt/β-catenin signaling pathway

Mitsuo Matsumoto, Naomi Ogawa, Tetsuya Fukuda, Yasuhiko Bando, Toshihide Nishimura, Jitsuo Usuda

**Figure S1.** Cluster dendrogram. The sixty protein network modules were identified by WGCNA analysis. The clustering feature seems to reflect a high similarity in protein expression but a difference only in their expression levels between A549 and A549/KL.

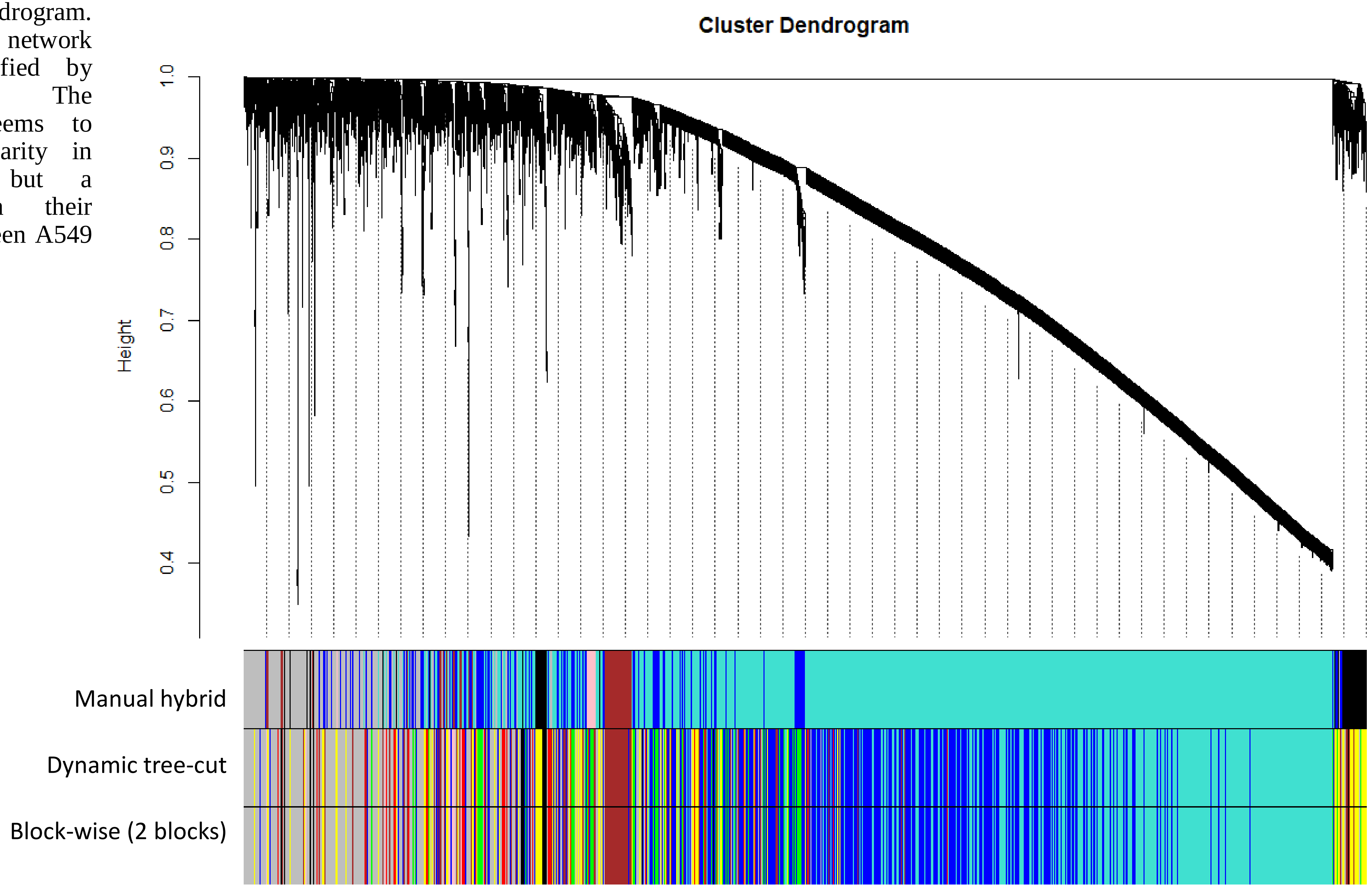

**Figure S2.** The module membership of module proteins (kME vs gene/protein significance) for three modules (WM55-2 (turquoise-2), WM55-3 (turquoise-3), and WM55-4 (turquoise-4)), which were obtained again by applying the 2nd WGCNA analysis to the WM55 (turquoise) significant to the A549/KL trait. The WM55-2 (turquoise-2) was by far significantly associated with the A549/KL trait, and total 582 proteins showed high gene/protein significance =1 and p-value < $1.0 \times 10^{-200}$.

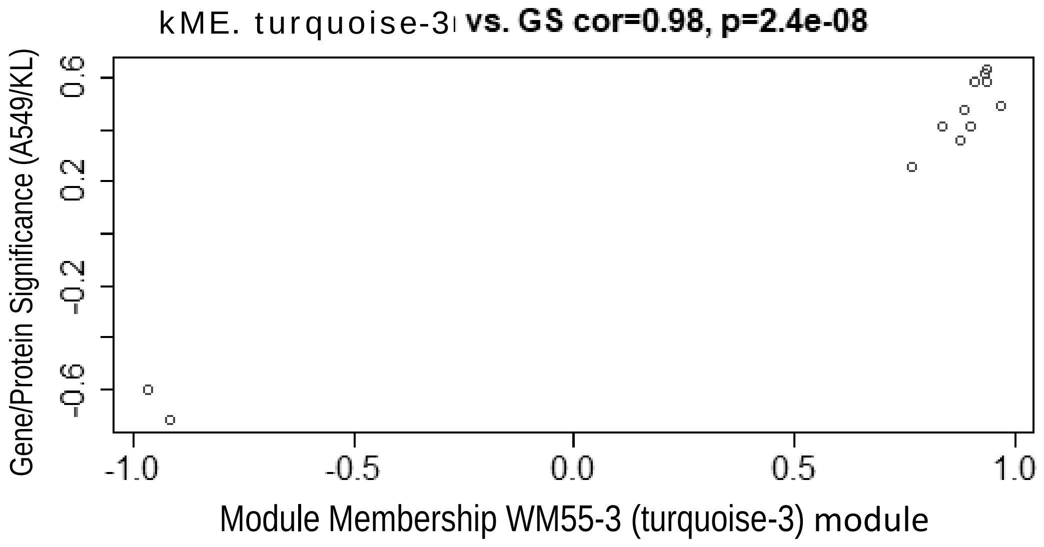


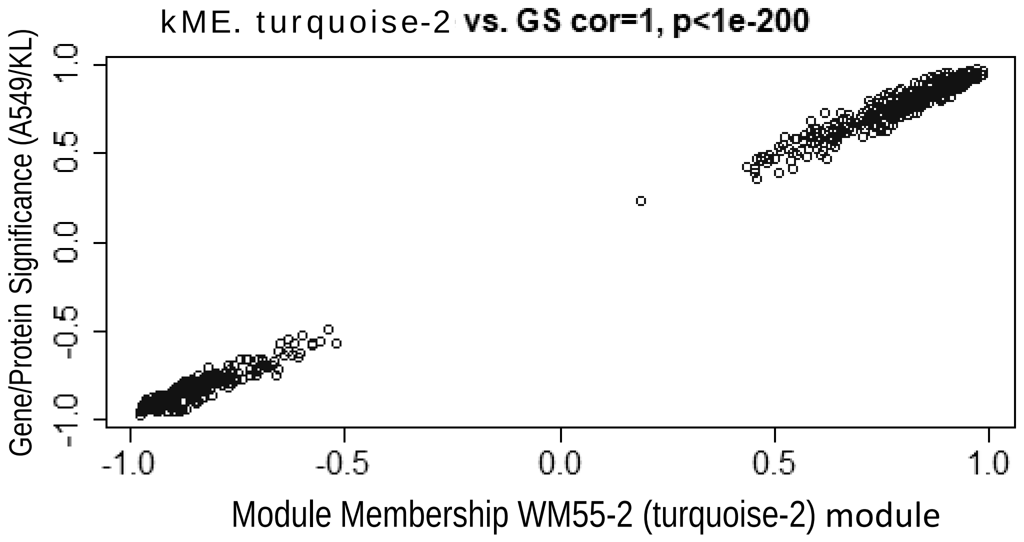


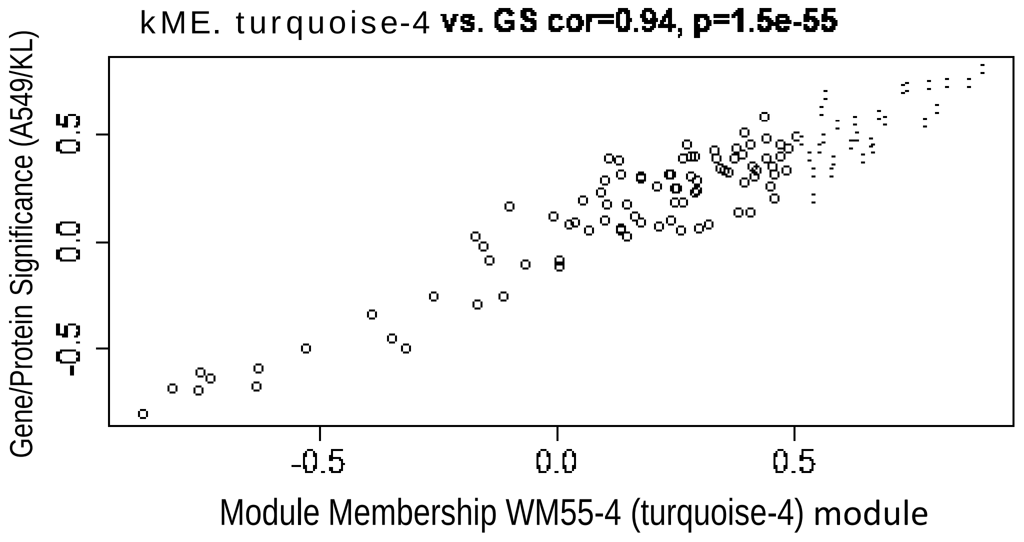

**Figure S3.** The PPI co-expression networks of the WM55-2 (turquoise-2) module (members *n* = 582).

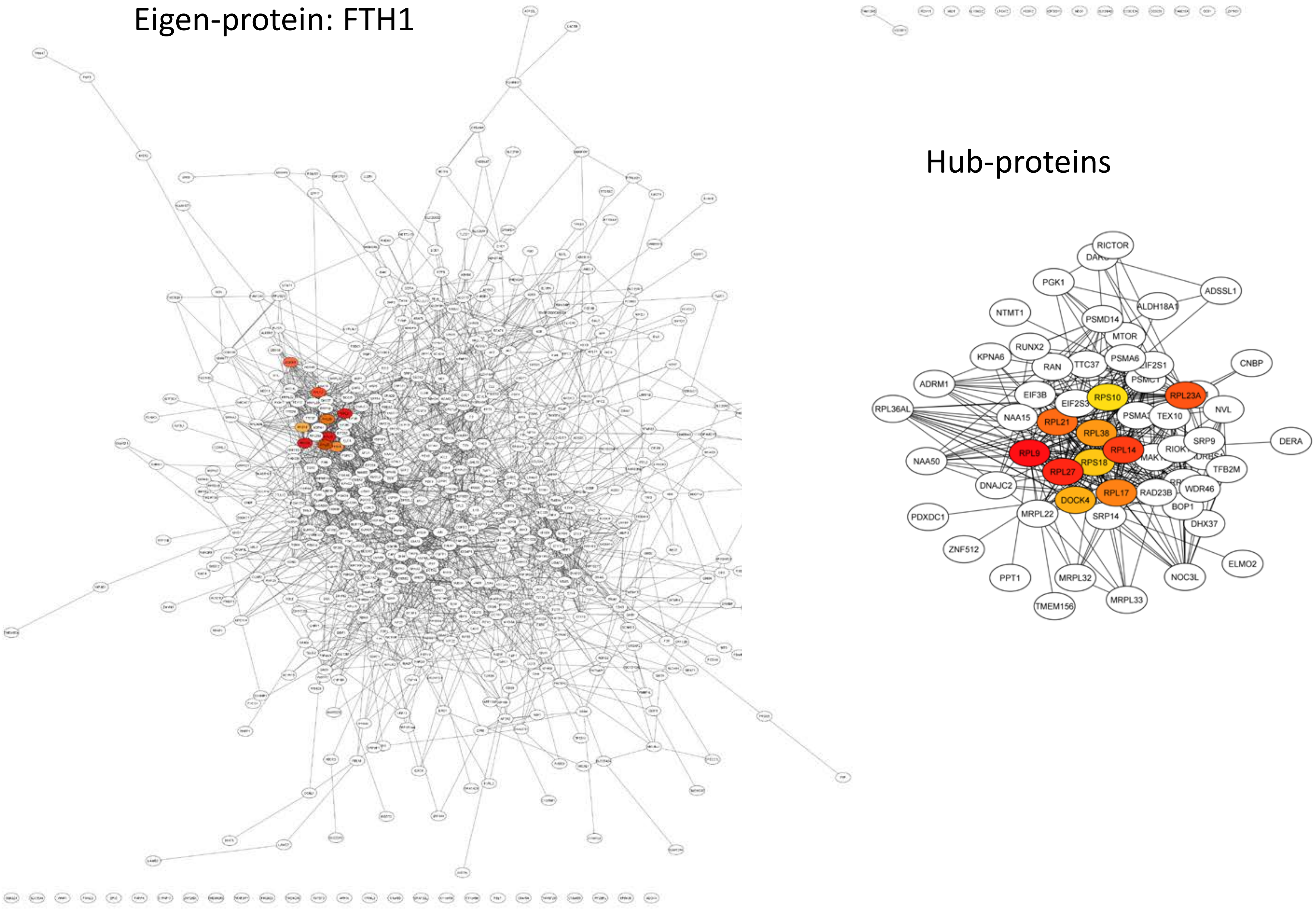

**Figure S4.** Integrative causal networks of Bim (BCL2L11) and Puma (BBC3) predicted significantly for the WM55-2 (turquoise-2) module, together with participating regulators, whereas targeting 139 proteins belonging to the module are not shown in this diagram.

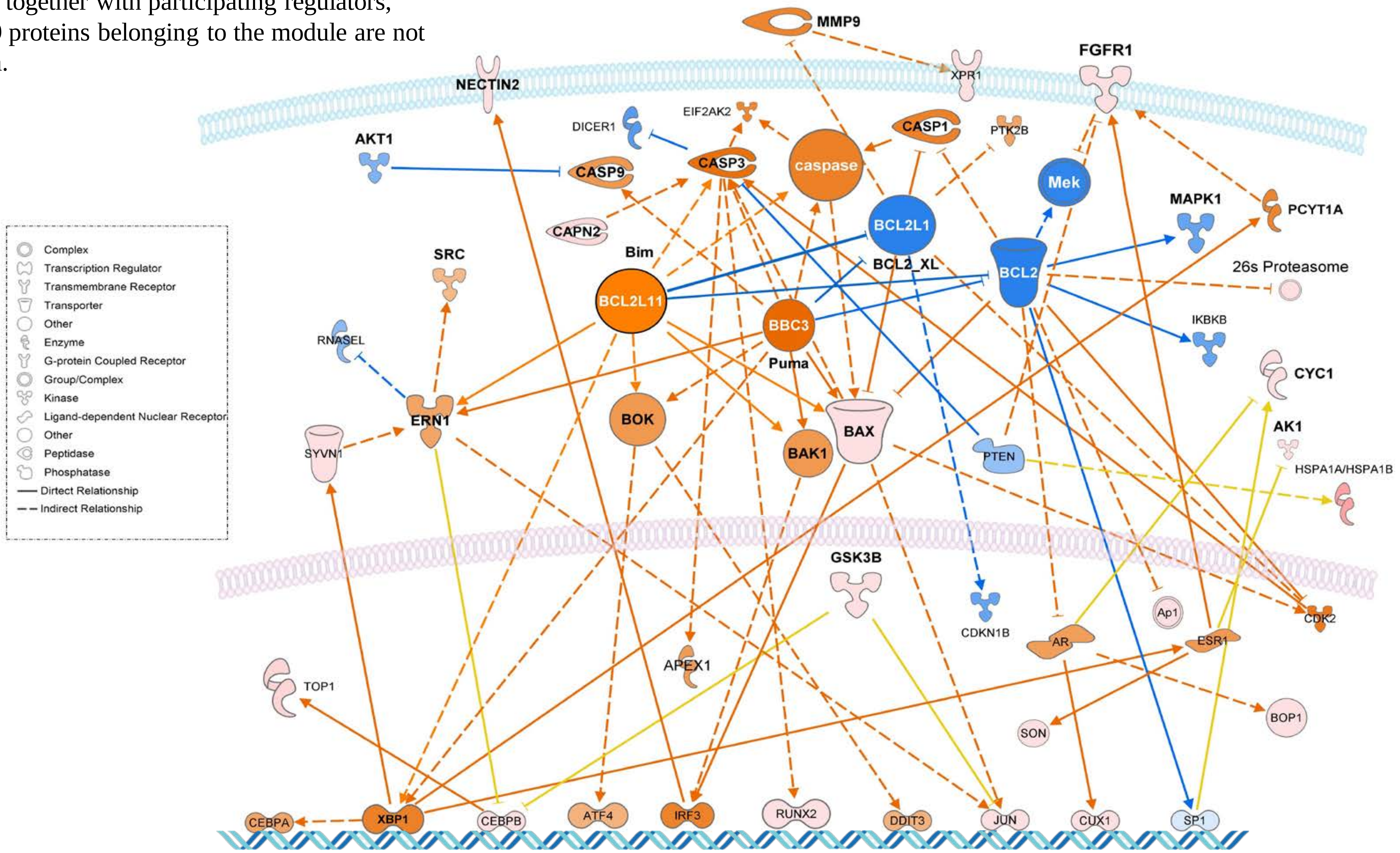

**Figure S5.** Kaplan-Meier plots for mRNA expressions of the key proteins. A) BCL2L11 (Bim), B) BOK, C) BCL2L1 (BLC2-XL), D) MCL1 (Myeloid Cell Leukemia Sequence 1 (BCL2-Related)), and E) CDH2 (N-cadherin).

The upregulated levels of both Bim (BCL2L11), BOK (BCL2 Related Ovarian Killer), and MCL1 (Myeloid Cell Leukemia Sequence 1 (BCL2-Related)) are associated with a better prognosis in OS in contrast to the upregulated BLC2L1 (BCL2-XL) with a poor prognosis. High expression of CDH2 (N-cadherin) correlated with relatively poor prognosis (OS).

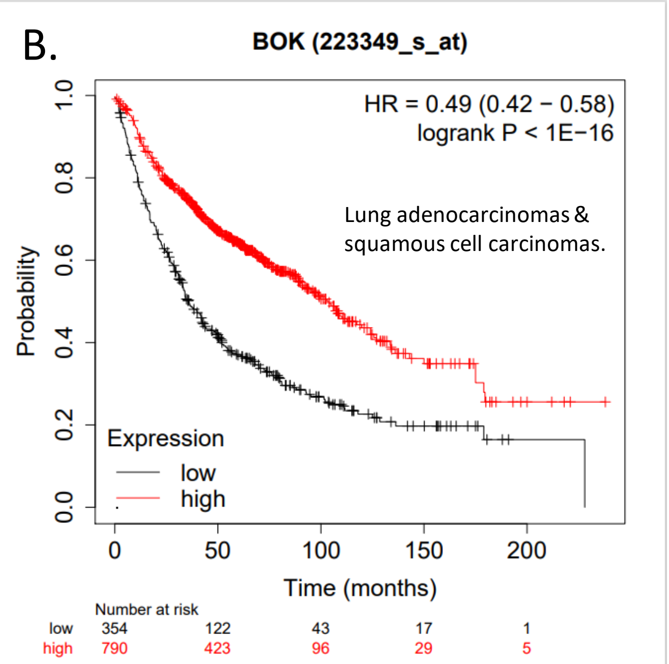


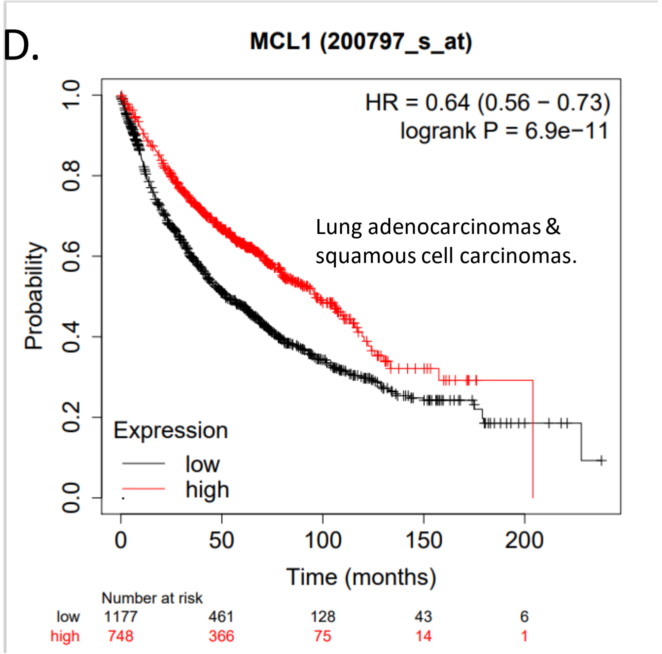


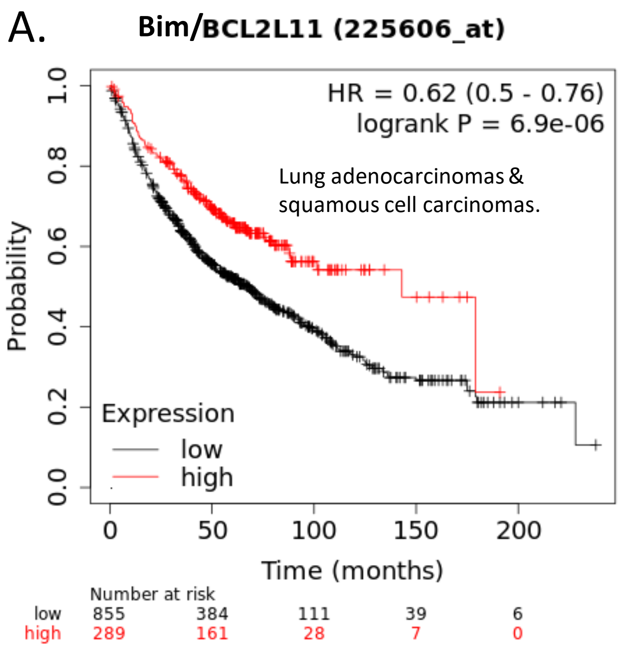


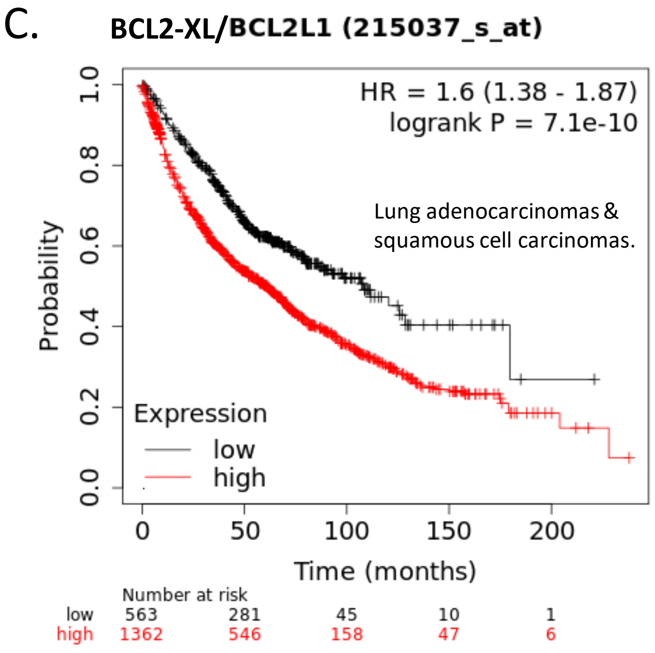


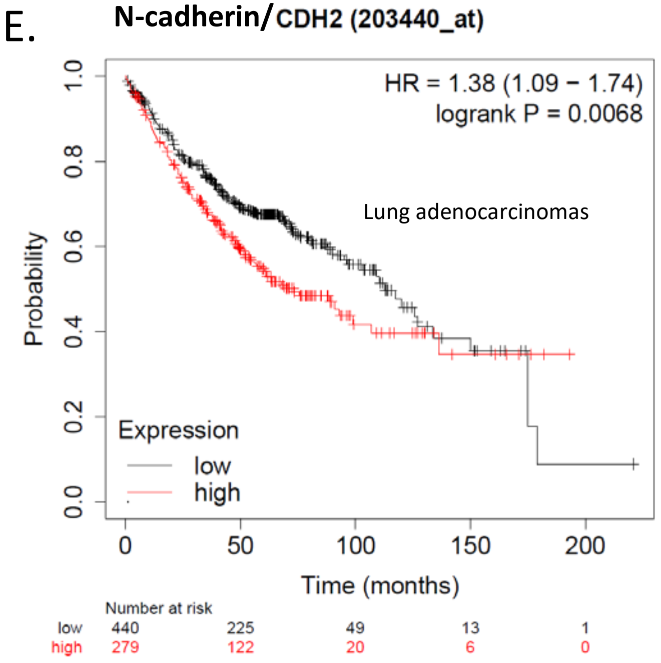

**Figure S6**. Gel images presented as full as possible length gels and blots with membrane edges visible for Klotho-GFP, Bim, Puma, β-Catenin, Phospho-β-Catenin (Ser675), Wnt5a and β-actin.

| | |
|---|---|
| 1 | ECL Rainbow Marker - Full Range |
| 2 | A549 |
| 3 | A549/KL |

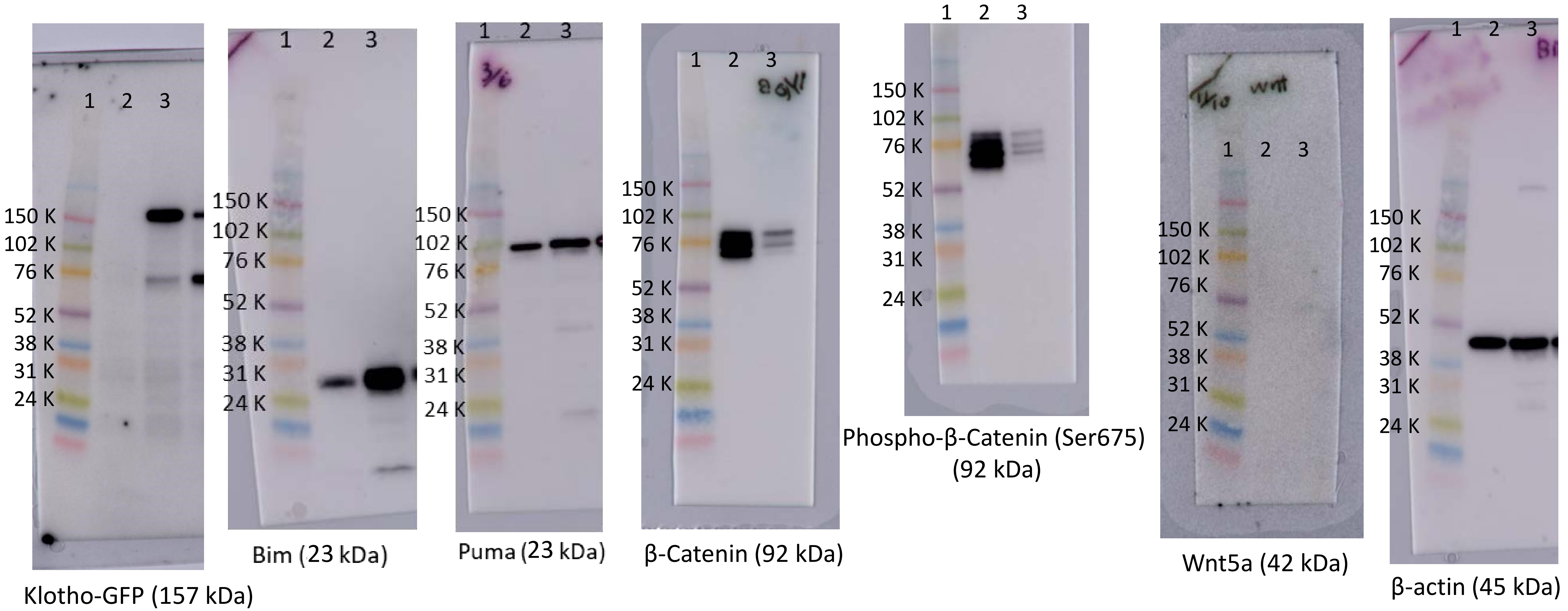

**Table S1.** The top 20 representatives of upstream master regulators (genes, RNAs, and proteins) (activated or inhibited: |z-value| > 2.0, and upregulated: 1.5 < z-value < 2.0) together with participating regulators, canonical pathways, and regulator effects are summarized for the WGCNA Turquoise-2 module. Activated NFE2L2 (master regulator of hypoxia), and co-activation of ERN1 and XBPI implicate the ER stress. The highly activated causal networks of both BBC3 (Puma) and BCL2L11 (Bim) were found only to include the key participating apoptosis regulator BOK, which was expressed uniquely in our proteomic analysis of A549/KL. A z-score in red and with a red background indicates activated and that in a blue and with a blue background inhibited.

| Top20 Upstream Regulators | Activation *z*-score | *p*-value of overlap | Top 20 Master Regulators & Causal Networks | Participating regulators | Activation z-score2 | *p*-value of overlap3 | Network bias-corrected *p*-value | Top 20 Canonical Pathways | *z*-score | *p*-value | Top 20 Regulator Effects: Diseases & Functions | Consistency Score |
|---|---|---|---|---|---|---|---|---|---|---|---|---|
| miR-124-3p (and other miRNAs w/seed AAGGCAC) | -4.74 | 1.01E-08 | miR-124-3p (and other miRNAs w/seed AAGGCAC) | miR-124-3p (and other miRNAs w/seed AAGGCAC) | -4.796 | 1.01E-08 | 1.00E-04 | Unfolded protein response | 1.89 | 7.079E-05 | Apoptosis of carcinoma cell lines,Apoptosis of lung cancer cell lines,Breast or pancreatic cancer,Cancer of cells,Cell viability of tumor cell lines,Familial congenital malformation,Growth failure or short stature,Growth of organism,Hypoplasia,Invasion of tumor cell lines,Migration of breast cancer cell lines,Migration of smooth muscle cells,Motor dysfunction or movement disorder,Organismal death,Synthesis of nucleotide | 37.324 |
| ERN1 | 2.26 | 2.77E-06 | CXCL14 | CXCL14,GABA,NFE2L2 | 6.301 | 9.93E-10 | 1.00E-04 | Huntington's Disease Signaling | 1.633 | 7.762E-05 | Apoptosis of carcinoma cell lines,Breast or pancreatic cancer,Cancer of cells,Cell movement of tumor cell lines,Cell viability of tumor cell lines,Familial congenital malformation,Growth failure or short stature,Hypoplasia,Invasion of cells,Migration of smooth muscle cells,Organismal death,Quantity of nervous tissue | 34.728 |
| miR-16-5p (and other miRNAs w/seed AGCAGCA) | -4.17 | 3.22E-06 | BBC3 | 26s Proteasome, AKT1,Ap1,APEX1,AR,ATF4,BAK1, BAX,BBC3,BCL2, BCL2L1,BOK,CASP1,CASP3,CASP9,caspase,CDK2,CDKN1B,CEBPA,CEBPB,DDIT3,DICER1,EIF2AK2,ERN1,ESR1,IKBKB,IRF3,JUN,MAPK1,Mek,MMP9,PCYT1A,PTEN,PTK2B,RNASEL,SP1,SRC,XBP1 | 4.835 | 1.61E-14 | 1.00E-04 | EIF2 Signaling | 2.333 | 0.0005 | Apoptosis of carcinoma cell lines,Breast or pancreatic cancer,Cancer of cells,Cell viability of tumor cell lines,Familial congenital malformation,Growth failure or short stature,Hypoplasia,Invasion of tumor cell lines,Migration of breast cancer cell lines,Migration of smooth muscle cells,Organismal death,Replication of virus | 30.111 |
| MYC | 3.55 | 3.26E-06 | RASAL1 | CEBPB,CREB1,EIF2AK2,EIF4E,ELK1,ESR1,HRAS,IKBKB,JUN,KRAS,MAP2K1/2,MAP2K5,MAPK14,MAPK7,MAPT,MMP9,MNT,MTOR,MYC,MYOD1,NFkB (complex),NFkB (family),NFKBIA,NRAS,Pkc(s),PTEN,PTGS2, PTK2B,Rap,RAS,RASAL1,RELA,SRF,STAT1,TSC2,YAP1 | -4.409 | 7.75E-17 | 1.00E-04 | Fatty Acid β-oxidation I | 2.236 | 0.0015 | Apoptosis of carcinoma cell lines,Breast or pancreatic cancer,Cell death of lung cancer cell lines,Cell viability of tumor cell lines,Dysgenesis,Familial congenital malformation,Growth failure or short stature,Growth of organism,Invasion of tumor cell lines,Migration of breast cancer cell lines,Migration of smooth muscle cells,Necrosis of liver,Organismal death,Proliferation of liver cells | 29.496 |
| EGFR | 2.50 | 6.75E-06 | Rasgrp | Akt,AKT1,Ap1,CEBPB,CHUK,CREB1,CXCL12,EIF2AK2,EIF4E,ERK,ERK1/2,ESR1,HRAS,IKBKB,ITGB1,JUN,KRAS,MAP2K1/2,MAP2K5,MAPK1,MAPK14,MAPK7,MAPK9,Mek,MMP9,MNT,MTOR,MYC,MYOD1,NCOR2,Nfat (family),NFkB (complex),NFkB (family),NFKBIA,NRAS,P38 MAPK,PI3K (complex),PIK3CA,Pkc(s),PTEN,PTGS2,RAF1,RAS,Rasgrp,RASGRP1,RASGRP2,RASGRP3,RELA,RPS6KB1,SRF,STAT1,STAT3,TSC2,YAP1 | 4.824 | 1.45E-16 | 1.00E-04 | NRF2-mediated Oxidative Stress Response | 2.646 | 0.0022 | Apoptosis of kidney cells,Chemotaxis,Dysgenesis,Growth failure or short stature,Invasion of breast cell lines,Invasion of tumor cell lines,Migration of breast cancer cell lines,Outgrowth of neurons,Proliferation of liver cells | 27.73 |
| NFE2L2 | 4.15 | 1.26E-05 | CEBPG | ACLY,Akt,CASP1,CDK2,CEBPB,CEBPD,CEBPG,ESR1,ESR2,FOXA1,H2AX,HIF1A,HNF4A,INSR,MYC,NFkB (complex),PI3K (complex),PPARG, RELA,TCF20,TCF3,TCF4,TFAP2A,transcription factor,XBP1,ZEB1 | -5.06 | 1.13E-15 | 1.00E-04 | CLEAR Signaling Pathway | 0.775 | 0.0047 | Apoptosis of lung cancer cell lines,Apoptosis of prostate cancer cell lines,Ataxia,Cellular degradation,Degeneration of cells,Invasion of tumor cell lines,Metabolism of nucleotide,Migration of smooth muscle cells,Necrosis of liver,Neoplasia of cells,Neurodegeneration,Organismal death | 23.219 |
| XBP1 | 3.99 | 1.86E-05 | GAS7 | Akt,AKT1,ANXA2,Ap1,APP,AR,CCND1,CD3,Cdc42,CDKN1B,CHUK,CREB1,CTNNB1,EGFR,EPHA2,ERBB2,ERK1/2,ESR1,FOXO3,GAS7,GSK3B,HRAS,IGF1R,IKBKB,Jnk,LCK,MAP2K1/2,MAPK1,Mek,MTOR,MYC,MYD88,NANOG,NFkB (complex),NFKBIA,P38 MAPK,PGR,PIK3R1,PTEN,PTK2, PTK2B,RAF1,ROCK2,SHC1,SNCA,SRC (family),STAT3,STAT6,TLR3,TLR4,TP53,TSC2,YAP1 | -4.857 | 1.34E-15 | 1.00E-04 | Epithelial Adherens Junction Signaling | 0 | 0.0054 | Cell death of breast cell lines,Cell viability of prostate cancer cell lines,Hypoplasia,Invasion of breast cell lines,Invasion of lung cancer cell lines,Migration of breast cancer cell lines,Outgrowth of neurons | 23.152 |
| IL4 | 5.26 | 8.57E-05 | PRRG4 | Akt,AKT1,ANXA2,Ap1,APP,AR,CCND1,CD3,Cdc42,CDKN1B,CHUK,CREB1,CTNNB1,EGFR,EPHA2,ERBB2,ERK1/2,ESR1,FOXO3,GSK3B,HRAS,IGF1R,IKBKB,Jnk,LCK,MAP2K1/2,MAPK1,Mek,MTOR,MYC,MYD88,NANOG,NFkB (complex),NFKBIA,P38 MAPK,PGR,PIK3R1,PRRG4,PTEN, PTK2,PTK2B,RAF1,ROCK2,SHC1, SNCA,SRC (family),STAT3,STAT6,TLR3,TLR4,TP53,TSC2,YAP1 | 4.941 | 1.43E-15 | 1.00E-04 | Role of PKR in Interferon Induction and Antiviral Response | -0.378 | 0.0065 | Apoptosis of kidney cells,Chemotaxis,Concentration of ATP,Dysgenesis,Growth failure or short stature,Invasion of tumor cell lines,Migration of breast cancer cell lines,Outgrowth of cells,Proliferation of liver cells,Proliferation of neural cells | 19.603 |
| TGFB1 | 3.76 | 1.31E-04 | BCL2L11 | 26s Proteasome,Ap1,APEX1,AR,ATF4,BAK1,BAX,BCL2,BCL2L11, BOK,CASP1,CASP3,caspase,CDK2,CEBPA,CEBPB,DDIT3,DICER1,EIF2AK2,ERN1,ESR1,IKBKB,IRF3,JUN,MAPK1,Mek,PCYT1A,PTEN,RNASEL,SP1,SRC,XBP1 | 4.906 | 3.94E-14 | 1.00E-04 | Stearate Biosynthesis I (Animals) | 2.236 | 0.0074 | Apoptosis of kidney cells,Colorectal tumor,Dysgenesis,Growth failure or short stature,Invasion of tumor cell lines,Migration of breast cancer cell lines,Necrosis of liver,Proliferation of liver cells | 19.402 |
| FN1 | 2.51 | 1.76E-04 | miR-16-5p (and other miRNAs w/seed AGCAGCA) | miR-16-5p (and other miRNAs w/seed AGCAGCA) | -4.243 | 3.22E-06 | 2.00E-04 | HER-2 Signaling in Breast Cancer | 1.897 | 0.0105 | Apoptosis of carcinoma cell lines,Apoptosis of kidney cells,Ataxia,Breast or colorectal cancer,Breast or pancreatic cancer,Cancer of cells,Cell death of lung cancer cell lines,Cell focus formation,Cell viability of tumor cell lines,Cleavage of glycosphingolipid,Colorectal tumor,Congenital neurological disorder,Early-onset neurological disorder,Endocytosis,Familial congenital malformation,Growth failure or short stature,Hypoplasia,Infection of cells,Invasion of tumor cell lines,Metabolism of sphingolipid,Migration of breast cancer cell lines,Migration of smooth muscle cells,Necrosis of liver,Neurodegeneration of Purkinje cells,Organismal death,Proliferation of liver cells,Synthesis of fatty acid,Synthesis of nucleotide | 17.591 |
| EIF2AK3 | 2.14 | 2.50E-04 | RAS | Akt,AKT1,Ap1,BRCA1,CEBPB,CHUK,CREB1,EIF2AK2,EIF4E,ELK1,ERK,ERK1/2,ESR1,HRAS,IKBKB,JUN,KRAS,MAP2K1/2,MAP2K5,MAPK1,MAPK14,MAPK7,MAPT,Mek,MNT,MTOR,MYC,Nfat (family),NFkB (complex),NFkB (family),NFKBIA,NRAS,P38 MAPK,PI3K (complex),PIK3CA,Pkc(s),PTGS2,RAF1,RAS,RELA,SRF,STAT1,STAT3,TSC2,YAP1 | 5.596 | 1.39E-13 | 2.00E-04 | Polyamine Regulation in Colon Cancer | 1.342 | 0.0145 | Apoptosis of carcinoma cell lines,Apoptosis of lung cancer cell lines,Cell viability,Hypoplasia,Invasion of cells,Necrosis of prostate cancer cell lines,Proliferation of fibroblast cell lines | 17.454 |
| NUPR1 | -2.35 | 2.55E-04 | Egfr-Erbb2 | 26s Proteasome,AGT,Akt,AKT1,ANXA7,Ap1,AR,CCND1,Cdc42,CDKN1A,CEBPB,Creb, CREB1,CTNNB1,EGFR,Egfr-Erbb2,EGR1,ELK1,EPB41,ERBB2,ERK1/2,ESR1,FOS,FOXO1,FOXO3, GATA3,GSK3B,GTP,HGS,HIF1A,HRAS,IKBKB,IL10,IRF3,KCNIP3,KRAS,MAP3K14,MAPK1,MAPK7,MED1,Mek,MMP9, MTOR,MUC1,MXD1,MYC,NCOA3,NFE2L2,NFkB (complex),NR4A1,NRAS, ODC1,PARP1,PDK1, PGR,PI3K (complex),PLCG2,PPARG,PTK2B,PTPN11,REL,RPS6KB1,RPTOR, SMAD3,SNCA,STAT3,STAT5B,STAT6,tamoxifen,TARDBP,TGFBR2,TSC2,USP8,WBP2,YAP1 | 4.569 | 2.83E-17 | 2.00E-04 | BEX2 Signaling Pathway | 0.816 | 0.0151 | Apoptosis of kidney cells,Cell death of breast cell lines,Cell viability of ovarian cancer cell lines,Cell viability of prostate cancer cell lines,Growth of neurites,Hypoplasia,Invasion of breast cell lines,Invasion of lung cancer cell lines,Migration of breast cancer cell lines,Outgrowth of cells,Proliferation of liver cells | 17.032 |
| CEBPB | 4.64 | 3.50E-04 | SHC-GRB2-GAB1 | 26s Proteasome,Akt,AKT1,Ap1,AR,CCND1,CDK2,CDKN1A,CEBPB,Creb, CREB1,ELK1,ERBB2,ERK1/2,ESR1,FOXO3,GAB1,GATA3,GRB2,HIF1A,HRAS,IKBKB,IL10,IRF3,ITGB1,KCNIP3,KRAS, MAP2K5,MAP3K14,MAP3K3,Mek,MTOR,MYC,NFE2L2,NFkB (complex),NFKB1,NOS2,NR4A1,NRAS,PDK1,PI3K (complex),PLCG2,PTEN,PTK2B,PTPN11, RAF1,RB1,REL,RELA,RPS6KB1,RPTOR,SGK1,SHC-GRB2-GAB1,SHC1,SMAD3,SP1,STAT3,TSC2 | 4.975 | 2.51E-15 | 2.00E-04 | AMPK Signaling | -1 | 0.0166 | Cell movement of tumor cell lines,Invasion of tumor cell lines,Lymphoreticular neoplasm,Myeloid or lymphoid neoplasm,Neoplasia of blood cells,Proliferation of liver cells | 16.333 |
| NORAD | 2.45 | 3.58E-04 | SP4 | Akt,AKT1,Ap1,BRCA1,CEBPB,CHUK,CREB1,EIF2AK2,EIF4E,ELK1,ERK,ERK1/2,ESR1,HRAS,IKBKB,JUN,KRAS,MAP2K1/2,MAP2K5,MAPK1,MAPK14,MAPK7,MAPT,Mek,MNT,MTOR,MYC,Nfat (family),NFkB (complex),NFkB (family), NFKBIA,NRAS,P38 MAPK,PI3K (complex),PIK3CA, Pkc(s),PTGS2,RAF1,RAS,RELA,SP4,SRF,STAT1,STAT3,TSC2,YAP1 | 5.657 | 8.55E-14 | 2.00E-04 | Aryl Hydrocarbon Receptor Signaling | 0.447 | 0.0166 | Apoptosis of carcinoma cell lines,Apoptosis of lung cancer cell lines,Apoptosis of prostate cancer cell lines,Cell viability,Hypoplasia,Necrosis of liver,Replication of virus | 15.057 |
| CD300LF | 2.24 | 3.63E-04 | IRS2-PI3K | 26s Proteasome,ADAM10,ADCY,AKT1,Ap1,AR,ATF6, CCND1,Cdc42,CDK2,CDKN1A, CEBPB,CREB1,CSF2RB,CTNNB1,EIF2AK3,EIF4E,ELK1,ERN1,ESR1,FOXO1,FOXO3,GATA3,HIF1A,HRAS,HSF1,IKBKB, IL10,Insulin,IRF3,IRS2,IRS2-PI3K,ITGB1,KCNIP3,KRAS,MAP2K5,MAP3K14, MAPK1,MAPK14,MAPK7,MEF2C,Mek,MKNK1,MMP9,MTOR,MYC,NFE2L2,NOS2,NR4A1,NRAS,P110,p85 (pik3r),PDK1,PI3K (complex),PI3K p85,PIK3C2A,PIK3CA,PIK3CB,PIK3CD,PIK3CG, PIK3R1,PIK3R2, PKD1,PLCG2,PRKCZ,PTK2B,RAF1,RB1,REL,RPS6KB1,RPTOR,SHC1,SLC2A4,SMAD3,SQSTM1,STAT6,STK11,TSC2 | 5.804 | 7.44E-15 | 3.00E-04 | Insulin Secretion Signaling Pathway | 3.606 | 0.0174 | Endocytosis,Hypoplasia,Invasion of carcinoma cell lines,Necrosis of liver | 13.796 |
| CNGA3 | -2.21 | 3.63E-04 | MKP1/2/3/4 | Akt,AKT1,Ap1,AR,ARRB1,CASP1,CCND1,CDKN1A,CDKN1B,CEBPA,CEBPB,Creb,CREB1,CTNNB1,DUSP1,DUSP4,DUSP6,DUSP9,EGFR,EGR1,ERK1/2,ESR1,ETV5,FOS,FOXO1,GATA1,GSK3B,HIF1A,IL10,ITGB1,JUN,MAPK1,MAPK7,MED1, MITF,MKNK1,MKP1/2/3/4,MMP9,MTOR,MYC,Nfat (family),NFE2L2,NFkB (complex),NFKBIA,NR3C1,NR3C2,PARP1,PPARA, PPARG,PTPRR,RAF1 ,RELA,RUNX2,Smad2/3, SMAD3,SP1,SRF,STAT3,STAT4,STK11,TCF3,TP53,TSC2,YBX1 | -5.344 | 1.22E-15 | 3.00E-04 | Xenobiotic Metabolism AHR Signaling Pathway | 0.816 | 0.0200 | Cell death of lung cancer cell lines,Dysgenesis,Necrosis of liver,Organismal death,Proliferation of liver cells | 13.152 |
| IL2 | 2.11 | 4.01E-04 | MPZL1 | AKT1,Ap1,AR,ARRB1,CASP1,CCND1,CD247,CD28,CD3,CDKN1A,CDKN1B,CEBPA,CEBPB,Creb,CREB1,CTNNB1,EGR1, ERBB2,ERK1/2,ESR1,ETV5,FOS,GATA1,HIF1A,HRAS,IL10,IRF3,ITGB1,JUN,MAPK7,MED1,MITF,MKNK1,MMP9,MPZL1,MTOR,MYC,NFE2L2,NR3C1,NR3C2,NRAS,PGR,PLCG2,PPARA,PTK2B,PTPN11,PTPRR,RAF1,RELA,SHC1,Smad2/3,SMAD3,SP1,SRF,STAT3,STAT5B,STK11,TCF3,TCR,TP53,TSC2,YBX1,ZAP70 | -5.514 | 7.62E-15 | 4.00E-04 | Xenobiotic Metabolism PXR Signaling Pathway | 1.897 | 0.0204 | Apoptosis of kidney cells,Cell death of breast cell lines,Endocytosis,Gliosis,Hypoplasia,Invasion of breast cell lines,Invasion of tumor cell lines,Migration of breast cancer cell lines,Proliferation of liver cells | 12.945 |
| E2F1 | 2.43 | 6.77E-04 | Vegfr dimer | 26s Proteasome,ADAM10,AKT1,Ap1,AR,CASP1,CCND1,CDK2, CDKN1A,CEBPB,CREB1,EGFR, ELK1,ERK1/2,ESR1,FLT1,FLT4,FOXO1,FOXO3,GATA3,GSK3B,HIF1A,IKBKB,IL10,IRF3,ITGB1,KDR,KRAS,MAP2K1/2, MAP3K14,Mek,MTOR,MYC,NFE2L2,NFKBIA,NOS2,NR4A1,NRAS,PDK1,PI3K (complex),PLC gamma,PLCG2,PTK2B,RAF1,RB1,REL,SHC1, SMAD3,STAT3,TSC2,Vegf,Vegf Receptor,Vegfr dimer | 6.368 | 4.07E-15 | 4.00E-04 | Pulmonary Fibrosis Idiopathic Signaling Pathway | 3.207 | 0.0309 | Apoptosis of tumor cell lines,Ataxia,Cellular degradation,Degeneration of cells,Neurodegeneration,Organismal death,Viral Infection | 11.25 |
| NPC1 | -2.95 | 7.03E-04 | miR-16-5p (and other miRNAs w/seed AGCAGCA) | ADAM10,ADCY,Akt,AKT1,BCL2,CASP1,CASP3,CCND1,CDKN1A,CEBPB,CHUK,Creb,CREB1,CTNNB1,EIF2AK2,ERK1/2,ESR1,FOXO1,GATA3,HIF1A,HRAS,IKBKB,IL10,IRF3,ITGB1,KCNIP3,KRAS,LPL,MAP2K1/2,MAP2K5,MAP3K14,MAPK1,MAPK14,Mek,miR-16-5p (and other miRNAs w/seed AGCAGCA),MTOR,MYC, MYOD1,NFE2L2,NFkB (complex),NFKBIA,NOS2,NR4A1 ,NRAS,PDK1,PI3K (complex),PLCG2,PTEN, PTK2B,RAF1,RB1,REL,SHC1,SMAD3,STAT1,STAT3,TSC2 | -6.746 | 5.52E-16 | 5.00E-04 | Regulation Of The Epithelial Mesenchymal Transition By Growth Factors Pathway | 2.333 | 0.0479 | Apoptosis of kidney cells,Cell death of breast cell lines,Cell viability of breast cancer cell lines,Chemotaxis,Concentration of ATP,Dysgenesis,Growth failure or short stature,Migration of breast cancer cell lines,Outgrowth of cells,Proliferation of liver cells,Proliferation of neuronal cells | 11.094 |
| let-7a-5p (and other miRNAs w/seed GAGGUAG) | -3.50 | 8.66E-04 | GABBR1 | ADCY,AKT1,Alp,AR,ARRB1,ATF3,ATF4,ATF6,CASP1,CCND1,CDK2,CDKN1A,CDKN1B,CEBPA,CEBPB,CREB1,CTNNB1,DDIT3,EGFR,EGR1,EPO,ERK1/2,ESR1,ETV5,EZR,F3,FOS,GABBR1,GATA1,GSK3B,HIF1A,IL10,IRF7,ITGB1,JUN,MAPK1,MED1,MITF,MKNK1,MMP9,MTOR,MYC,Nfat (family),NFE2L2,NFkB (complex),NFKBIA,NR3C1,NR3C2,P2RY2,PGR,PLC,PLCG2, PPARA, PPARG,PRL,PTPRR,RAF1, RELA,RUNX2,SELP,Smad2/3,SMAD3,SP1,SRF,STAT3,TCF3,TP53,TSC2,YBX1 | 4.916 | 3.71E-15 | 5.00E-04 | EGF Signaling | 2 | 0.0479 | Hypoplasia,Necrosis of liver | 11 |
